# Choice- and trial-history effects on causality perception in Schizophrenia Spectrum Disorder

**DOI:** 10.1038/s41537-025-00614-0

**Published:** 2025-04-17

**Authors:** Kai Streiling, Rasmus Schülke, Benjamin Straube, Loes C. J. van Dam

**Affiliations:** 1 Institute for Psychology and Centre for Cognitive Science, Technical University of Darmstadt, 64283 Darmstadt, Germany; 2 Department of Psychiatry, Social Psychiatry and Psychotherapy, Hannover Medical School, 30625 Hannover, Germany; 3 Center for Mind, Brain and Behavior, University of Marburg and Justus Liebig University Giessen, 35032 Marburg, Germany; 4 Department for Psychiatry and Psychotherapy, Philipps University Marburg, 35039 Marburg, Germany; 5 Department of Psychology, University of Essex, Colchester, UK

**Keywords:** Schizophrenia, Human behaviour

## Abstract

Perceiving causality is a low-level, immediate cognitive process based on temporal and spatial cues relating to sensory events and could be viewed as a perceptual judgement. Perceptual judgements in general are affected by a choice- and trial history bias, however, it is not yet fully understood how such a bias integrates into the perception of causality. Here, we investigate judgements of perceptual causality in Schizophrenia Spectrum Disorder (SSD) as a perceptual decision process with systematic influences from past choices and experiences. We analysed previously collected data from a causality-judgement experiment using Michotte launching events and examined differences between patients with SSD (SSDs) and healthy control participants (HCs). We did this on several levels to shed light on known dysfunctions in the judgement of cause-effect relations in SSD, such as the jumping-to-conclusions bias. Using multiple Generalized Linear Mixed-Effects Models (GLMMs) revealed a significant direct influence of the choice-history for both participant groups. Trial-history (previous stimulus experiences) on the other hand appears to exert a more subtle influence on the current choice by modulating the effect of choice-history and current spatial and temporal properties. Regarding the stimulus of a given trial, SSDs relied more on spatial properties and less on temporal properties than HCs. Furthermore, an analysis of effects across time suggested an increasing reliance on previous choices for SSDs, and a decreasing effect for HCs. This hints towards a potentially maladaptive pattern which might contribute to biased causal attributions in SSD.

## Introduction

We humans are causal creatures that constantly search for connections between events to make sense of the world around us^1^. This compulsion leads us to even *see* causality between sensory events when there is no physically plausible causal interaction. Albert Michotte was the first to study this perception of causality as a low-level, immediate process using animations of colliding objects^2^. He manipulated these collisions, for example by adding an angle to the movement of the second object relative to the first. Michotte showed that while participants reported perceiving less causality for higher angles the amount of causal responses was still significant. This suggests that our perception of cause-effect relations accounts not only for physical plausibility but likely incorporates uncertainties about the environment or our senses or includes the spatial or temporal context of the current event. This perception and judgement of causality has been reported to be disturbed in patients with Schizophrenia Spectrum Disorder (SSD)^3,4^. However, the influencing factors of this typical perturbation are still not fully understood. In this study we aim to disentangle the effects of spatial and temporal features and short-term biases on perceptual causality judgements and their differences in SSD.

Since Michotte, such colliding-object stimuli, coined the Michotte Launching Task, have frequently been used to further investigate the perception of causality^3,5–7^ and its relation to the perception of spatial or temporal features^8,9^.

Bechlivanidis and Lagnado, for example, reported that the expected causal relation influences the temporal order in which events are remembered^8^. Deeb et al. showed that causal priming can influence the perception of apparent motion^9^. Mayrhofer and Waldmann asked participants not only whether an event showed a causal relation but to identify the source object of the interaction^6^. Observing launching events between two objects A and B, participants had to report which of the objects was initiating the change, e.g. “A launched B” or “B stopped A”. They called this object the *causal agent*, a term that had often been used in developmental studies^10,11^, and found that the attribution of causal agency is independent of the perception of forces.

Straube and Chatterjee investigated the relation between temporal and spatial attributes on the perception of causality using animated launching events^5^. Varying delays and angles of egress (the angle between the movement of the first and second object) they underlined that causality is perceived for a wide range of spatial and temporal violations of continuity. Moreover, using functional magnetic resonance imaging (fMRI) they identified brain regions linked to spatial and temporal processing as being involved in these judgements of perceptual causality. Follow up studies using tDCS^12^ showed that a tDCS modulation of the influence of spatial violations was stronger for trials with more ambiguous temporal information.

These results suggest that, i) participants’ percepts and judgements of causality are partially based on cues that go beyond the presented stimulus, such as expectations or prior experiences and, ii) that perception of causality and perception of space and time in the physical world influence each other.

In Schizophrenic Spectrum Disorder (SSD) this process of causal attribution can be disturbed^4^. A misperception or misattribution of causality may be connected to delusion symptoms or materialise as a “jumping-to-conclusion” bias. While this bias is connected to prediction and uncertainty evaluation mechanisms, it may also arise if links on a chain of arguments are skipped because their causal relation is misperceived. Tschacher and Kupper, for example, used a variation of the collision event stimulus, the stream-bounce illusion, in which two objects move towards each other and when overlapping continue to move in the same direction. This can be perceived as the objects streaming through/behind each other (stream-percept, non-causal) or bouncing off each other (bounce-percept, causal). They showed that positive symptoms in SSD patients relate to increased perceived causality when shown a stream-bounce illusion while disorganisation symptoms correlated with attenuated causality perception.

Moreover, it has been shown that patients with SSD show a longer temporal binding window^13–16^. This implies that they have a higher tolerance to delay between events which could be due to impaired access to temporal information^17^. Thus, we can expect SSDs to generally rely less on temporal features for perceiving causality and more on other cues that for instance carry spatial information.

As many perceptual decision-making tasks, the above-mentioned experiments generally assume that judgements are only based on the current stimulus. It has however been shown that perceptual decisions also depend on a context that is shaped by past decisions^18–20^, the confidence in those decisions^19^, and the recent history of experiences, i.e. the history of stimuli on previous trials^21^. These history biases can either increase or decrease the proportion of causal judgements. Adaptation effects to delay^22,23^ for example can lead to a higher proportion of causal judgements. Effects like the location dependent orientation aftereffect of tilted lines^24,25^ on the other hand can lead to less causal judgements. A similar location-specific aftereffect was observed for the stream-bounce illusion^26^. After an adaptation phase of observing repeated stream-bounce events (unbiased in their spatio-temporal features (no delay or angle of egress) and with minimal overlap) in a certain part of the visual field, participants judged subsequent events in that part of the visual field as less causal than in another, non-adapted location. These findings underline the view of causality as an immediate, low-level (visual) percept and suggest that visual adaptation effects can occur in locally distinct parts of the visual field.

The underlying mechanisms of these biases are not yet fully understood^21^ and it remains a challenge to distinguish between low-level aftereffects on perception itself and effects on the reported judgement^25^. There is consensus that perceptual judgements of causality have a low-level perceptual and a mid- or high-level cognitive component^26,27^ and that trial-history effects could be expected on both levels^25^. However, to our knowledge there are no studies investigating their interplay in this domain and their effects in SSD. Previous work has described altered^28^, increased^29^ and unchanged^30^ reliance on prior information in perceptual decision making in SSD. Potential aberrations of priors might translate to history biases as well. Given known issues in processing temporal cues, we might expect SSDs to compensate not only by relying more on spatial features but also on contextual information such as their previous choice or previous stimuli.

**Fig. 1:**
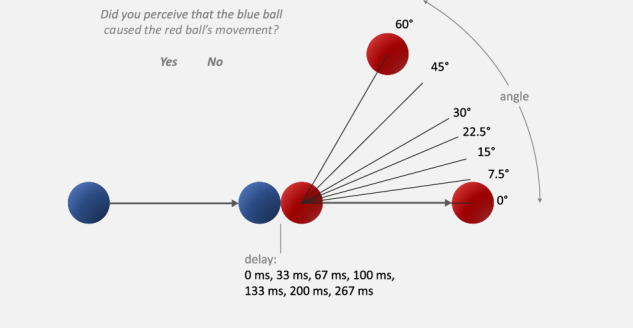
Schematic procedure of one trial. The blue ball starts at one side of the screen (either left or right) and moves towards a red ball positioned towards the middle of the screen with constant velocity. Upon collision, the blue ball stops and the red ball commences movement after a certain delay and at a certain angular offset in movement direction^7^.

In this study we reanalysed data from a previously conducted experiment^7^ and broke down the influence of recent experiences into a trial-history and a choice-history bias. Figure 1 shows a schematic of a trial displaying a typical Michotte Launching Event with different angles of egress and delays. Participants were asked to report their intuitive and immediate percept of causality when judging whether the first ball launched the second or not. We fitted Generalised Linear Mixed-Effects Models (GLMMs) using Bayesian statistics and tested the hypothesis that neurotypical participants and participants with SSD differ with regard to their use of spatial and temporal information and their choice- and trial-history biases. As expected, we found SSDs to rely more on spatial and less on temporal features. However, there was no generally higher choice- or trial-history effect for SSDs. Instead the development of this bias differed between groups. The next section will present the results of our analysis, followed by a discussion and details on the experiment and models.

## Results

We fitted Generalised Linear Mixed-Effects Models (GLMMs) using angle *α*, delay *δ*, participant group *g*, previous choice *c*_1_ and the spatial and temporal properties of the previous trial *α*_1_ and *δ*_1_ as well as interaction effects between these parameters as described in Section “Models”. Table 1 shows the posterior distributions of the parameters estimated by the GLMM defined in Eq. (3).

**Table 1:** Posterior parameter distributions.

Parameter *β*_*X*_	Pre stimulation				Post stimulation			
	Mean	SD	CI 3%	CI 97%	Mean	SD	CI 3%	CI 97%
0 (Intercept)	−0.369	0.175	−0.679	−0.036	−0.308	0.170	−0.619	0.025
*g* (group)	0.030	0.176	−0.289	0.367	0.119	0.174	−0.226	0.440
*α* (angle)	**−0.580**	0.021	−0.619	−0.539	**−0.703**	0.023	−0.749	−0.661
*δ* (delay)	**−0.842**	0.022	−0.887	−0.803	**−0.943**	0.024	−0.989	−0.900
*c*_1_ (prev. choice)	**0.194**	0.021	0.156	0.234	**0.260**	0.023	0.214	0.300
*α*_1_ (prev. angle)	0.010	0.021	−0.030	0.049	0.046	0.023	0.004	0.087
*δ*_1_ (prev. delay)	0.028	0.021	−0.011	0.066	−0.000	0.022	−0.042	0.040
*g* : *α*	**−0.141**	0.022	−0.179	−0.098	**−0.226**	0.023	−0.268	−0.183
*g* : *δ*	**0.324**	0.023	0.280	0.365	**0.374**	0.024	0.330	0.420
*g* : *c*_1_	**−0.062**	0.022	−0.102	−0.022	0.015	0.022	−0.027	0.057
*g* : *α*_1_	**−0.049**	0.021	−0.088	−0.008	−0.031	0.022	−0.071	0.010
*g* : *δ*_1_	**−0.094**	0.021	−0.132	−0.053	−0.061	0.022	−0.102	−0.019
*c*_1_ : *α*	**0.081**	0.021	0.042	0.121	**0.037**	0.023	−0.008	0.078
*c*_1_ : *δ*	**0.095**	0.022	0.053	0.134	**0.072**	0.023	0.028	0.115
*g* : *c*_1_ : *α*	**−0.063**	0.021	−0.105	−0.024	**−0.072**	0.023	−0.112	−0.028
*g* : *c*_1_ : *δ*	−0.040	0.022	−0.081	0.002	−0.047	0.023	−0.092	−0.005
*α* : *c*_1_ : *α*_1_	**0.448**	0.023	0.402	0.489	**0.352**	0.024	0.307	0.396
*g* : *α* : *c*_1_ : *α*_1_	**0.219**	0.023	0.173	0.260	**0.135**	0.024	0.087	0.179
*δ* : *c*_1_ : *δ*_1_	**0.266**	0.022	0.225	0.306	**0.277**	0.024	0.234	0.324
*g* : *δ* : *c*_1_ : *δ*_1_	−0.016	0.022	−0.057	0.025	−0.033	0.024	−0.077	0.013

Selected parameters of the GLMM defined in Eq. (3) fitted separately on all data before and after tDCS. Reported are estimated posterior distributions, characterised by posterior mean, standard deviation (SD) and the 94% Bayesian credibility interval (CI) (also referred to as highest density interval (HDI)) with 3% and 97% thresholds. Reporting 94% CI was chosen to avoid confusion with significance testing that sometimes uses a 5% threshold. See also ref. ^51^. Parameters with a mean in bold are considerably different from zero, that is they do not contain zero in the reported HDI and can be considered real influences on participant’s responses according to the model. For a full list of model parameters and further discussion of the interaction-effects of higher order see Supplementary Material B. For an alternative analysis of the main effects using a Frequentist approach see Supplementary Material F.

Note that the original experiment^7^ included tDCS stimulations between two blocks of measurement which was not the focus of the current study (though see Supplementary Material D for a reanalysis of the effects of tDCS confirming previous observations). The remainder of this section therefore largely presents pre-stimulation parameter fits only, except for an analysis investigating trends over longer periods of time where we used both pre- and post-stimulation data (see Section “Parameter development over time”). We confirmed that tDCS did not influence the main findings reported therein (see Supplementary Material D).

Perceptual judgements of causality were mainly influenced by the spatial (*α*) and temporal (*δ*) properties of the present trial (*β*_*α*_ < 0 and *β*_*δ*_ < 0 in Table 1) for both healthy control participants (HCs) and participants with SSD (SSDs). In both groups, higher angles and delays led to lower proportions of “causal” responses.

Also the previously made choice (*c*_1_) had a considerable binding effect (*β*_*c*1_ > 0), i.e. if the previous response was “causal”, participants were more likely to report “causal” again on the current trial.

The direct influence of spatial and temporal properties of the previous trial (*α*_1_ and *δ*_1_) on the other hand was not substantial (*β*_*α*1_ and *β*_*δ*1_). Using model comparison, however, we found that only including choice- and trial-history effects *and* their interactions improved model fit (see Supplementary Material A for more details).

These interaction-effects of higher order (*β*_*α*:*c*1:*α*1_ and *β*_*δ*:*c*1:*δ*1_) suggest a strong interdependence between the current trial’s spatio-temporal properties and choice- and trial-history.

The two groups differed in several behavioural aspects: (i) Within a single trial, HCs are more influenced by delay while SSDs base their judgements more on the angle of egress (*β*_*g*:*α*_ and *β*_*g*:*δ*_). (ii) The choice-history bias decreased for HCs but increased for SSDs over the course of each block (pre- and post-stimulation). (iii) The current trial’s spatial and temporal properties modulated the choice-history binding slightly differently for HCs and SSDs (see *β*_*g*:*c*1:*α*_ and *β*_*g*:*c*1:*δ*_): Ambiguity in the preferred property (angle for SSDs and delay for HCs) led to stronger reliance on the previous choice.

### Effects of spatial and temporal cues

Delay and angle both have a negative effect on causality judgements (*β*_*α*_ = −0.580, 94% CI = [−0.619, −0.539] and *β*_*δ*_ = −0.842, 94% CI = [−0.887, −0.803]). This means that withincreasing angle between the incoming an the outgoing ball’s movement (angle of egress) and increased delay between the “hit” and the movement onset of the second ball, the movement of the second ball is less often perceived as being caused by the incoming ball.

**Fig. 2:**
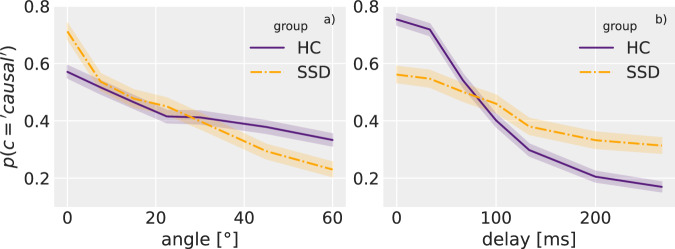
Effects of spatial and temporal cues on reported causality. The probability of reporting causal *p*(*c* = *causal*) depending on angle (panel **a**) and delay (panel **b**), split for HCs and SSDs for the pre-stimulation data. The graphs show the mean and 95% confidence intervals calculated using non-parametric bootstrapping as implemented by the seaborn python package^52^. SSDs show a stronger dependence on spatial properties and a weaker dependence on temporal properties than HCs when reporting perceived causality. A similar effect for the post-stimulation sessions is shown in Supplementary Material B.

In line with previous research^3^, SSDs and HCs differ in their use of spatial and temporal information. With increasing angle (Fig. 2 left side), the decline in reporting perceived causality is steeper for SSDs compared to HCs. With increasing delay (Fig. 2 right side), the decline is less steep for SSDs compared to HCs. These differences are reflected in the group-angle interaction effect *β*_*g*:*α*_ (mean = −0.141, 94% CI = [−0.179, 0.098]) and the group-delay interaction effect *β*_*g*:*δ*_ (mean = 0.324, 94% CI = [0.28, 0.365]) shown in Table 1. We can translate these interaction effects into group specific effects by combining them with the base effect (*β*_*α*_ and *β*_*δ*_) as shown in Eq. (1).

1 $$\beta_{\alpha,g}=\beta _\alpha +g\,{{\cdot }}\,\beta _{g:\alpha}$$

SSDs (coded as *g* = 1), thus, are more influenced by spatial features (*β*_*α,g*=′*SSD*′_ = −0.72, *β*_*α,g*=′*HC*′_ = −0.44) and less influenced by temporal features (*β*_*δ,g*=′*SSD*′_ = −0.52, *β*_*δ,g*=′*HC*′_ = −1.17) compared to HCs (coded as *g* = −1). These effects remain consistent in the post-stimulation data (see Supplementary Material B).

### Direct choice-history effects

Additionally to spatial and temporal information, the previous choice has a significant influence on perceptual causality judgements. If we split the choice-history parameter *β*_*c*1_ (mean = 0.194, 94% CI = [0.156, 0.234], see Table 1) into separate effects for each group based on the interaction effect *β*_*g*:*c*1_ (mean = −0.062, 94% CI = [−0.102, −0.022]) (equivalent to Eq. (1)), we get a choice-history effect of 0.256 for HCs (*β*_*c*1*,g*=′*HC*′_ = 0.256, 94% CI = [0.202, 0.307]) and 0.132 for SSDs (*β*_*c*1*,g*=′*SSD*′_ = 0.132, 94% CI = [0.071, 0.19]). These effects are positive, which means that if one reported “causal” (“non-causal”) in the previous trial, one is more likely to report “causal” (“non-causal”) again in the current trial.

As further described below (see Section “Parameter development over time”), the choice-history bias of SSDs shows a strong development over time. While in the beginning of a session HCs appear to be more influenced by their previous choice than SSDs, the bias grows stronger for SSDs over time.

### Ambiguity in trial properties modulates choice-history effect

**Fig. 3:**
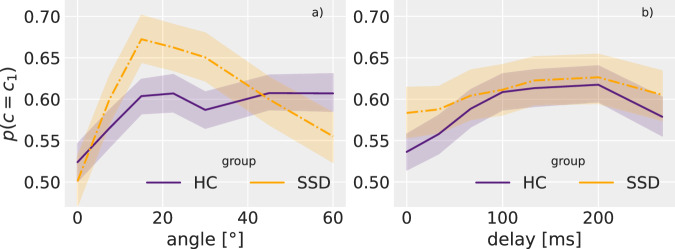
Effects of spatial and temporal cues on repeating an answer. The probability of reporting the same as in the previous trial *p*(*c* = *c*_1_) depending on angle (panel **a**) and delay (panel **b**), split for HCs and SSDs for pre-stimulation data. (For a similar figure containing post-stimulation data see Supplementary Material B). The graphs show the mean and 95% confidence intervals calculated using non-parametric bootstrapping as implemented by the seaborn python package^52^.

Additionally to a direct choice-history effect, the model suggests an interaction between the choice-history and the current trial’s spatial and temporal properties (see parameters *β*_*c*1:*α*_ (mean = 0.081, 94% CI = [0.042, 0.121]) and *β*_*c*1:*δ*_ (mean = 0.095, 94% CI = [0.053, 0.134]) in Table 1). Figure 3 displays the choice-history binding effect represented by the probability of giving the same judgement as in the previous trial (*p*(*c* = *c*_1_)) on the y-axis against the denormalised values of angle and delay on the x-axes.

The relation we observe is clearly non-linear and, thus, only partly described by a GLMM. When we assume linearity, the parameter *β*_*c*1:*α*_ > 0 (*β*_*c*1:*δ*_ > 0) suggests that with higher angle (delay) a perceptual judgement depends more on the previous choice. However, examining the underlying data in Fig. 3 we find that the influence of the previous choice is strongest for non-extreme values of angle and delay. Values at the extremes of the provided range (0–60° and respectively 0–250 ms) lead to a decreasing choice-history binding (*p*(*c* = *c*_1_) closer to 0.5). These findings suggest that non-extreme angles and delays (in the scope of the presented stimuli range) present more ambiguity in terms of causality which is compensated by a stronger choice-history bias.

In the spatial domain (Fig. 3, left side) SSDs show a strong modulation, while there is no such effect for HCs. In the temporal domain, both groups show this choicebinding modulation through delay (Fig. 3, right side), however, it is more pronounced for HCs and weaker than in the spatial domain for SSDs. These group differences are reflected by the model in the three way interaction effects *β*_*g*:*c*1:*α*_ and *β*_*g*:*c*1:*δ*_ (see Table 1).

### Interplay of choice- and trial-history effects

We found that including both, choice- and trial-history effects *and* their interactions, leads to an improved model fit (see Supplementary Material A for the full GLMM model comparison). While the direct trial-history effects of angle and delay are neglectable (see *β*_*α*1_ and *β*_*δ*1_ in Table 1), their interaction effects with the choicehistory and their current trial’s counterpart (*β*_*α*1:*c*1:*α*_ and *β*_*δ*1:*c*1:*δ*_) are considerable. Furthermore, the model reports a substantial group difference of this interaction-effect for angle (*β*_*g*:*α*1:*c*1:*α*_).

**Fig. 4:**
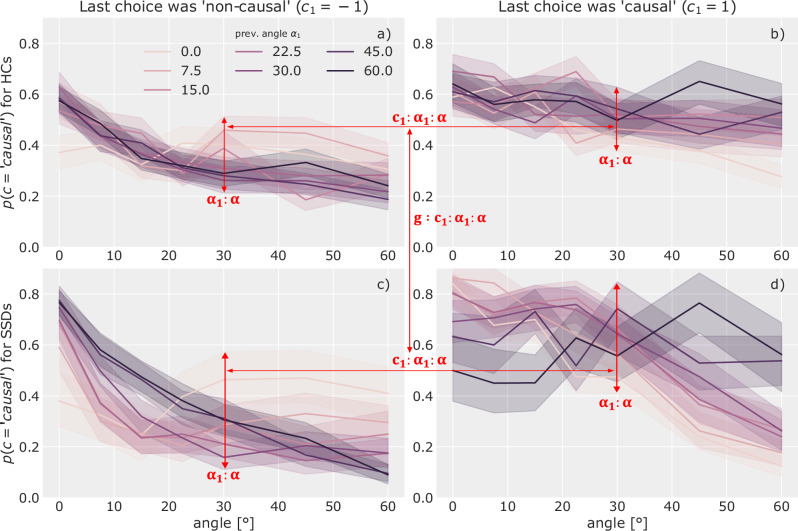
Angle and choice-history interactions. The figure shows the influence of the current trial’s angle *α* on the probability to report perceiving causality *p*(*c* =^′^ *causal*^′^). In each panel, this relation is split according to the value of the angle on the previous trial *α*_1_ (colour coded). The leftside panels (**a**, **c**) only show trials where the previous response was “non-causal” and the right-side panels (**b**, **d**) only those for which it was “causal”. The top row (**a**, **b**) displays data of HCs, the bottom row (**c**, **d**) of SSDs. The shaded areas represent confidence intervals calculated using bootstrapping^52^. Arrows indicate where interaction-effects can be observed in the data. For example, the spread between the coloured lines visualises how the previous angle modulates the effect of the current angle on reporting causality. The difference in this spread between the left and right side panels visualises how the previous response changes this *α*_1_ : *α* modulation.

**Fig. 5:**
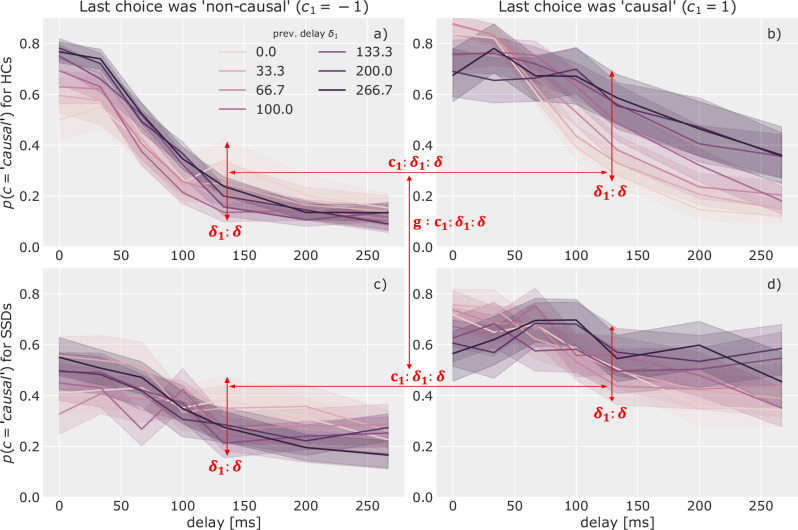
Delay and choice-history interactions. The figure shows the influence of the current trial’s delay *δ* on the probability to report perceiving causality. Within a panel, this influence is split according to the value of the previous delay *δ*_1_. Furthermore, the relation is shown separately for trials for which the previous response was “non-causal” (left side, **a**, **c**) and for which it was “causal” (right side, **b**, **d**) and for HCs (top row, **a**, **b**) and SSDs (bottom row, **c**, **d**). The shaded areas represent confidence intervals calculated using bootstrapping^52^. Arrows indicate where the interaction-effects can be observed in the data.

Figure 4 shows the data underlying these effects in the spatial domain, Fig. 5 displays data for the temporal domain. They display the influence of the current trial’s angle (delay) on the probability to respond “causal”, separately for each possible angle (delay) on the previous trial. The components of the interaction-effects are marked in red to help localize them in the presented data. The remainder of this section will describe and interpret these effects in detail.

Each panel in Fig. 4a–d shows the interaction-effect between the angle-history and the current angle (*β*_*α*1:*α*_) in the spread and relation of the different lines which encode the previous trial’s angle. Looking at panel a), we see that when the previous angle *α*_1_ was 0° *and* the previous choice *c*_1_ was “non-causal”, the current trial’s angle has only a *weak* influence on the perceptual causality judgement. Reporting “non-causal” in the previous trial despite observing a 0° angle is most likely driven by a non-spatial cue, presumably delay. Participants might carry over this focus on non-spatial features to the current trial which reduces the influence of angle.

Panel b shows the same relation when the previous choice was “causal”. Here, the interaction is reversed and we find a reduced angle-effect when the previous angle was high, which is inline with the above explanation. This inversion of the *α*_1_ : *α* interaction effect depending on the reported choice is captured in the parameter *β*_*α*1:*c*1:*α*_ (mean = 0.448, 94% CI = [0.402, 0.489]). A corresponding pattern is found for delay (see Fig. 5).

In the spatial domain the model reported a substantial difference of above described interactions between SSDs and HCs through the parameter *β*_*g*:*α*1:*c*1:*α*_ (mean = 0.219, 94% CI = [0.173, 0.260]). This difference can be found by comparing the top row (HCs) and bottom row (SSDs) in Fig. 4. For both possible previous choices (left and right side) the spread between lines increases visibly for the SSD group.

First, for SSDs the current angle had a significantly stronger influence on the reported percept compared to HCs when the previous choice was most likely made based on the angle too. Specifically, when the previous angle was high and the previous choice was “non-causal” (dark lines in panel c)) also the current angle had a strong influence (stronger slope). Similarly, the current angle also had a strong influence, when the previous angle was low and the previous choice was “causal” (bright lines in panel d)).

Second, in the opposite case, i.e. when the previous angle was 0° but the choice was “non-causal” for SSDs (bright line in panel c)), the current angle’s effect is reduced. When the previous choice was “causal” even though the previous angle was high (dark line in panel d)), the current angle’s effect seems to even be inverted: A higher angle leads to a higher probability of reporting “causal”. A potential cause of this behaviour lies in the reduced reliance on timing information in SSDs: If previously an angle of 60° was perceived as “causal”, the current decision is more likely only influenced by delay, as by design any possible angle of the current trial is lower than 60° and, thus, falls in the angle range currently perceived as more “causal”. The influence of delay, however, is generally weaker for SSDs (see Fig. 2), which might lead participants to instead rely on heuristics such as the choice-history bias.

Similar trends can be observed for the temporal domain, by focusing on the interactions between the delay of the current and the previous trial and the choice made on the previous trial (*β*_*δ*1:*c*1:*δ*_ = 0.266, 94% CI = [0.225, 0.306]). When the previous delay was small, yet the response “non-causal”, the response on the current trial was less influenced by the current delay. Similarly, if the delay was at its maximum on the previous trial (15 frames or ca. 266 ms), yet the response “causal”, the influence of the current delay was also reduced. This effect seems somewhat stronger for HCs compared to SSDs, likely because HCs generally relied more on delay when making the responses. Note however, that though the trends in the interactions follow the same logic for the delay as for the angle of egress, the difference between HCs (top row in Fig. 5) and SSDs (bottom row in Fig. 5) was not substantial according to the model’s interaction parameter *β*_*g*:*δ*1:*c*1:*δ*_ (mean = −0.016, 94% CI = [−0.057, 0.025]).

### Parameter development over time

**Fig. 6:**
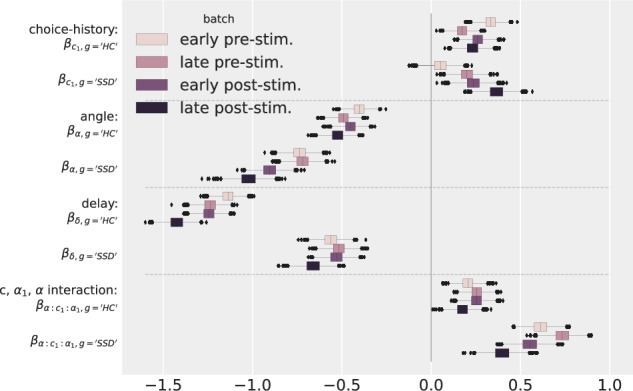
Parameter development. Posterior distribution of parameters split by participant group (HC and SSD) for each of the four batches of data in the experimental sessions (pre-stimulation and post-stimulation blocks each split in two to identify trends over time irrespective of tDCS stimulation). The reported parameters are the sum of the base parameters and the respective interaction. For example, *β*_*c*1_*,g* = *β*_*c*1_ + *g*·*β*_*g*:*c*1_, *g* = 1 if SSD, *g* = −1, if HC. Each box shows the mean and the 25% and 75% quartile. The whiskers include datapoints within 1.5 times IQR (inter-quartile range). Any points outside are drawn as dots. Positive values indicate that with higher values of the respective independent variable a “causal” response is more likely.

Taking a look at the post-stimulation parameters in Table 1, we often observe a slight shift compared to their pre-stimulation fit. In order to further analyse such potential changes over time in the main parameters and their differences between HCs and SSDs, we further split the pre- and post-stimulation data into four batches (early and late pre-stimulation and early and late post-stimulation), as described in Section “Models”, and fitted the full interaction GLMM defined in Eq. (3) separately on these data batches. The resulting most interesting parameter developments are reported in Fig. 6. For a complete list of parameters see Supplementary Material C. The top row shows the development of the choice-history bias. During each block (pre- and post-stimulation) the bias slightly decreased for HCs (*β*_*c*1*,g*=′*HC*′_ is smaller in the “late” batches than in the “early” batches) but clearly increases for SSDs (*βc*_1_*,g* = ′*SSD*′).

The middle rows display the parameters of the influence of the current trial’s spatial and temporal properties on perceptual causality judgements (*β*_*α*_ and *β*_*δ*_). The model suggested an increasing influence of angle and delay for HCs during both blocks. This development is slightly stronger for delay than for angle of egress, which again might be connected to delay being the more influential cue for HCs. SSDs show no consistent parameter development within the pre- and post-stimulation blocks. Between blocks, however, we can observe a jump in the angle effect which suggests a tDCS effect. To set parameter developments apart from tDCS effects, we fitted a GLMM including stimulation parameters and did point-wise data comparison. We could replicate the findings in ref. ^7^, namely a significant tDCS effect of fronto-parietal stimulation (left-frontal cathodal to right-parietal anodal) on the angle-effect for SSDs: angle variations in movement direction had a stronger effect on causality judgments after fronto-parietal stimulation. However, we found no other significant effects of tDCS (see Supplementary Material D for more details). These results are in line with previous accounts of parietal stimulation changing causality perception through influencing the processing of spatial information^31^. Yet they cannot confirm findings of the effect of frontal stimulation.

For trial-history parameters *β*_*α*1_ and *β*_*δ*1_ we found no clear development (see Supplementary Material C for an extended figure). The only other considerable change over time can be observed in the angle, choice- and trial-history interaction *β*_*α*:*c*1:*α*1_ for SSDs shown in the bottom row of Fig. 6. While increasing in the pre-stimulation block, after tDCS it decreases, which again hints towards a tDCS effect on the angle-influence for SSDs.

## Discussion

This study investigated how choice-history (previous trial’s response) and trial-history (previous trial’s spatial and temporal stimuli) together with the current trial’s angle and delay influence perceptual causality judgements in simulated launching events. We found an atypical pattern in these effects for patients with schizophrenia spectrum disorder (SSD) when compared to healthy control participants (HCs), which might contribute to biased causal attributions in SSD.

We found a considerable choice-history bias to repeat the same answer as on the previous trial for both groups. This bias developed differently for HCs and SSDs over the course of one session: For HCs it slightly diminishes while SSDs show an increase. An influence of past decisions on current perceptual decision making had already been reported for other modalities^18–20^. Furthermore, we found a potential maladaptive behaviour in the development of this bias for SSDs. HCs might use their previous choice as an initial heuristic and over time learn the scope of the task so they rely more and more on spatial and temporal information present in the current trial. This could, for example, be observed as an increase in parameter strength of the angle and delay influence for HCs in Fig. 6. SSDs may have problems integrating past experiences to learn the scope of the spatial and temporal features and instead rely more and more on short-term strategies such as their previous choice as a useful heuristic (top row Fig. 6). The absence of an increase of delay and angle effects for SSDs during the pre-stimulation block, as was observed for HCs, supports this hypothesis. It is worth mentioning that SSDs showed no increased fatigue or systematic tendency to repeat an answer irrespective of the stimulus towards the end of a session compared to HCs. This adaptation might also be similar to an effect previously reported for ambiguous stimuli^32^: The absence of feedback leads to a self-reinforcement of an intrinsic bias, in this case, the choice-history bias.

The previous trial’s angle and delay on the other hand did not exert a considerable *direct* influence on current perceptual judgements. However, we found that the effect of the current trial’s spatial and temporal properties depended on the combination of the respective previous trial’s property and the corresponding previous response. Taken together, these findings suggest that participants base their judgement also on the relative difference between the previous and the current trial put into context by their previous choice. If the previous choice was likely ignoring one cue as it carried little or insufficient information this cue’s effect is reduced in the current trial. If, for example, one reported “no causality” despite observing an angle of 0°, this decision is likely based on a non-spatial cue, e.g. delay. In this case, the angle cue was not informative for the decision and its weight in the current decision is decreased. On the other hand, a cue’s effect is increased if it was informative in the previous trial. For example, if one reported “no causality” at a 60° angle, the angle cue’s influence increases. This effect might occur at the interception of low-level and higher-level perception. The previous choice could set or shift the starting point of the current percept (similar to what has previously been modelled using Drift-Diffusion models^20^), shifting the same values for angle or delay towards being perceived as more or less causal. This three-way modulation furthermore substantially differed between participant groups in the spatial domain: SSDs showed a stronger modulation, i.e. the previous angle together with their previous choice had a stronger influence on their evaluation of the current angle. Future work should take this finding as indication to have longer breaks between trials, so this potentially perceptual after-effect may wear off.

SSD is often linked to a disturbed temporal processing and impaired access to timing information in general^17^ which materializes as a longer unisensory^14–16^ and multisensory temporal binding window^13,33–35^. Here, we found evidence that SSDs are less reliant on temporal information (delay) when judging perceived causality compared to the control group (see Fig. 2), which could result from above described impairments. SSDs might compensate the reduced certainty about temporal aspects in the stimulus by a stronger reliance on spatial features as well as a stronger reliance on choice-history.

As mentioned above, the increased reliance on choice-history could be due to a selfreinforcement process in the absence of feedback and otherwise ambiguous conditions. Our results support this idea when looking at the modulation of the choice-history effect by the current trial’s angle and delay. As reported in Fig. 3, the probability of repeating an answer depends on the ambiguity of the presented spatial and temporal cues. For HCs this effect is visible only in the temporal domain, for SSDs the modulation is specifically pronounced in the spatial domain. This could point towards a compensatory mechanism using choice-history as a fall-back cue, when current information in the stimulus is ambiguous. While stimuli were rendered with a 3D effect to look more realistic, the background and physics were unrealistic (grey background, linear object movement without acceleration and no bounce-off effect). It has been shown that visual realism of the whole scene (without realistic physics) weakens overall causality ratings^36^. The stimuli used in this study are closer to the original, simple Michotte stimuli than a realistically rendered scene (see video in Supplementary Material), making such global effects unlikely to play a role. This lack of stimulus realism can also be a source of ambiguity. Reversing the conclusion by Meding et al., less realistic looking stimuli may come with less expectation for realistic physics^36^ and thus more uncertainty about object characteristics (e.g. spin) and interaction rules. We find further support for this theory in existing work that has previously reported problems in decision making under ambiguity in patients with SSD^37^. Such problems could be linked to difficulties in interpreting ambiguous stimuli^38^. Further studies might explore this hypothesis of erroneous self-reinforcement.

### Limitations

This study and the findings presented herein come with some limitations which we sum up in the following.

First, the underlying experiment was designed to explore tDCS effects on perceptual causality^7^. While we found no considerable tDCS effects other than what was previously reported and performed additional analyses to assert no effects on our reported findings, we cannot fully rule out residual stimulation effects on the parameter developments reported here.

Second, the relatively small number of healthy participants, which was matched to the number of patients, also means that inference to a general population should be taken with a grain of salt. It would for instance be meaningful to correlate the observed effects reported here with the symptom strengths of individual participants even among HCs. However, since among patients symptom scores varied little and such scores were not recorded for HCs, this correlation analysis was not meaningful with the current dataset.

Third, as already indicated, the presented stimuli moved linearly and showed no realistic physical behaviour. This introduced room for interpretation and ambiguity. The effect of more realistic physics and varying plausible interaction settings, i.e. varying collision angles, should be explored in future studies. Also, while causality has long been investigated as an all-or-nothing concept, giving binary answers may have obscured insecurity and perceptual ambiguity. A rating or scale-based estimate could improve the investigation of uncertainty and cue-ambiguity.

Fourth, viewing distance was not fixed and participants were not shown fixation points. Instead they could freely view the collision events. This might have imposed inconsistencies between trials and likely increased the noise in the responses due to the potentially varying gaze positions. Such an effect might have been stronger for SSDs since patients might show impaired smooth pursuit^39,40^. However, given the repeated and prolonged experimental sessions, which already involved an experimental procedure possibly perceived as uncomfortable (tDCS), maximizing patient comfort and avoiding drop outs was a major concern, which led to the decision of not using the chinstrap.

Fifth, our analysis only included one-step-back effects. It would be of interest to also, for example, investigate the potential effects of long chains of same answers to see if the history bias accumulates. Such an investigation would however require an experiment designed with this question in mind such that longer sequences of same answers are more likely and is therefore better left as a future direction.

Furthermore, GLMMs are a powerful statistical method that helped to reveal conceptual influences and interactions of choice- and trial-history. Yet, naturally they fall short of explaining the underlying mechanisms. We can only take the reported parameters as a starting point to speculate about the processes that cause them.

Finally, based on this study alone it is impossible to discriminate effects on relatively low-level or high-level cognitive processes (i.e. basic visual perception or inferential reasoning). These issues of interpretation are shared by all similar investigations of perceptual causality^41^. Future studies could include established measures of low-level processing (e.g. P300), to determine whether the manipulations affect more basic or higher level cognitive processes.

## Conclusion

Our results underline a disturbed processing of temporal features for SSDs which appears to be compensated by stronger reliance on spatial features and choice-history. For SSDs, the choice-history bias increased over the course of an experimental session, which presents us with another reason to keep measurement sessions with patients short. This maladaptive process could impair patients in long, repetitive tasks that require attention, regular decisions and consideration of given sensory information. Our findings suggest to expect a significant role of choice- and trial-history biases and specifically their interactions in other perceptual decision making domains as well, even more so for patients with SSD. Especially ambiguity due to an absence of feedback may lead to erroneous self-reinforcement.

## Methods

This report reanalyses data collected by Schu¨lke, Schmitter and Straube^7^. While the initial analysis focused on the effects of tDCS in patients with SSD, we here analyse choice-history and trial-history effects on the causality judgement task in patients with SSD (SSDs) and healthy controls (HCs). The data on HCs was not published previously. In the following, an overview of the main experimental methods is provided (for a full description of the protocol we refer to the original paper^7^). After a summary description of the experiment, we will present a description of the statistical analysis and models particular to the present work.

### Experiment

20 patients with SSD were recruited at the Department of Psychiatry and Psychotherapy, Philipps-University Marburg, Germany. Demographic and basic neurocognitive data were collected. Diagnoses of SSD were made according to the International Statistical Classification of Diseases and Related Health Problems, Version 10, German Modification (ICD-10 GM). 28 participants without SSD (healthy controls not previously reported) were additionally recruited via posters placed in public buildings in Marburg or email-lists at the University of Marburg.

The experiment was approved by the local ethics committee (Ethik-Kommission des Fachbereichs Medizin der Philipps-Universit¨at Marburg) and was performed in accordance with the Declaration of Helsinki, except for pre-registration. Informed consent was obtained from all participants.

Data collection took place on several days. On a single day, a participant performed two times the same 98 trials in pseudo-randomized order. Between these two measurement blocks transcranial Direct Current Stimulations (tDCS) was applied with electrodes placed in one of four different combinations of cortical areas: (i) left and right parietal, (ii) left frontal and right parietal, (iii) left and right frontal and (iv) a sham stimulation on left frontal and right parietal. For tDCS the DC-STIMULATOR PLUS hardware (neuroConn GmbH) was used. Each participant went through all stimulations in a pseudo-randomized order. Each stimulation was applied on a different day with at least 20 h between two sessions to ensure after-effects had worn off. A current of 1.5 mA was applied to the scalp using saline-soaked sponges placed on rubber electrodes which resulted in a current density of 0.043 mA/cm². The stimulation was applied for 10 minutes. As a control condition, the sham stimulation applied only two brief stimulations in the form of two sinus half-waves with a ramp-up and ramp-down time of 15 s and a maximum current of 1.5 mA at the beginning and end of the 10 min stimulation phase to mimic stimulation onset and offset.

In each block (one before and one after stimulation), participants were shown 98 video clips in pseudo-randomized order depicting a launching event as described in Fig. 1. Each stimulus had a duration of 2 s, a resolution of 720 × 576 pixels, a framerate of 60 Hz, and displayed two round objects (a blue and a red “ball”) which were shaded to add pictorial depth cues (created with Strata 3D software, Corastar, Inc. dba Strata Software). Each trial initially showed a stationary blue and red ball. The blue ball started moving horizontally 400 milliseconds after the start of the trial and it came in contact with the red ball after 1000 milliseconds. At that moment, the blue ball stopped and the red ball started moving away from the blue ball with some delay (0, 2, 4, 6, 8, 12, or 16 frames which approximately translates to 0, 33, 67, 100, 133, 200, or 267 milliseconds) and angle (0, 7.5, 15, 22.5, 30, 45, or 60 degrees). Both objects moved with the same constant speed of 5 cm/s in all trials. The combination of these seven delays and seven angles resulted in 49 different movements which were shown once left-to-right and once right-to-left yielding above mentioned 98 trials per block and in total 16 repetitions per video per participant across sessions. Stimuli were presented with a viewing distance of approximately 60 cm and under free-viewing conditions. A constant distance could not be enforced due to some of the patients’ conditions. The used monitor had a diagonal of 60.5 cm, which resulted in each ball having a diameter of 1.6 cm and a distance covered by one ball of 4.6 cm.

Participants were tasked to judge perceived causality of the launching event (“Left click if you believe that the blue ball caused the movement of the red ball. Right click if you don’t believe the blue ball caused the movement of the red ball.”). It was made clear that there were no right or wrong answers, instead emphasis was placed on reporting their “immediate, individual, intuitive perception” as fast as possible. Furthermore, they were informed about possible delays and angles in advance and they were told that both balls would always come into contact with each other. An explicit instruction on how to rate causality was not provided. After the instructions, participants conducted several training trials. For each trial the given response (‘causal’ or ‘non-causal’) and the response times were measured.

### Data processing

For analysing choice-history and trial-history biases in perceptual judgements of causality we defined multiple Generalized Linear Mixed-Effects Models (GLMM) using the Python libraries Bambi (v0.12.0)^42^ and PyMC (v5.6.1)^43^ and fitted them with the provided implementation of Markov chain Monte Carlo (MCMC) sampling with the No U-Turn Sampler (NUTS).

For balancing the two groups of healthy control participants (HCs) and participants with SSD (SSDs) a subset of 20 individuals was selected from the recorded 28 HCs to demographically match SSDs. (See Table 6 in the Supplementary Material for a more detailed group comparison.) One SSD and one HC participant were excluded, because they exclusively or almost exclusively reported perceiving causal events (‘causal’ response rate of 100% and 98.3%).

The angle of egress *α* and the delay *δ* were normalized with mean 0 and standard deviation 1 for better parameter comparability and more robust convergence. The reported choice *c* and the participant group *g* were each encoded as sum-contrast^44^ (*c*: ‘non-causal’: −1 and ‘causal’: 1. *g*: ‘HC’: −1 and ‘SSD’: 1). To include choice- and trial-history biases, angle, delay and choice were shifted forward per measurement block and added to the following trial as *α*_1_, *δ*_1_ and *c*_1_ respectively.

This leaves us with six independent fixed-effects variables: angle *α*, delay *δ*, participant group *g*, previous choice *c*_1_ and the spatial and temporal properties of the previous trial *α*_1_ and *δ*_1_. We investigated the influence of these variables on the current choice *c*, the dependent variable in the models described below.

### Models

To estimate the effects of the above-described variables on the perceptual causality judgement we used Generalized Linear Mixed-Effects Models (GLMM), which have been applied in psychophysical^7,45^ and visual perception tasks^46^. Using sampling-based Bayesian statistics GLMMs enable us to also analyse uncertainties of the reported weights and, by adding random-effects, we can further segregate this uncertainty into a part inherent to the effect (fixed-effects) and another based on individual differences (random-effects). Here, we modelled the probability of reporting ‘causal’ *p*(*c*_*t*_ = *causal*) as a linear combination of weighted features passed through a non-linear link function *f*. Assuming a Bernoulli distribution, we used the standard logistic function (also referred to as *expit* function, see Eq. (2)).

2 $$f\left(x\right)=\frac{1}{1+\exp (-x)}$$

Initially, we fitted a GLMM including all above mentioned features as well as their interactions as fixed-effects and an intercept per participant as random-effect as shown in Eq. (3) (in lme4-style notation as described in ref. ^47^).

3 $$c\sim g\times \delta \times \alpha \times c_1\times \delta _1\times \alpha_ 1+(1\left|{subj}\right.)$$

This translates to a linear combination of features with weights *β* as indicated in Eq. (4) for a single time step *t*. The ellipses stand for the interaction terms following from Eq. (3). In total, the model has seven main effects (one intercept plus one parameter for each feature), 57 interaction effects, and 38 random-effects which are the participant specific intercepts (19 HCs and 19 SSDs).

4 $$\begin{array}{c}P({c}_{t}={causal})=f({\beta }_{0}+{\beta }_{g}{g}_{t}+{\beta }_{\delta }{\delta }_{t}+{\beta }_{\alpha }{\alpha }_{t}\\ \qquad\quad+\,{\beta }_{c1}{c}_{t-1}+{\beta }_{\alpha 1}{\alpha }_{t-1}+{\beta }_{\delta 1}{\delta }_{t-1}+\ldots )\end{array}$$

The model was fitted separately on data before (8 trials per condition) and on data after tDCS (8 trials per condition) resulting in two sets of parameters reported in Table 1. Fitting was done using 4 chains with 2000 samples per chain where the first 1000 samples were used for tuning. Tuning or burn-in samples are used to allow a Markov Chain to reach its equilibrium distribution before collecting samples for parameter estimation. Priors were weakly informative Normal distributions with mean zero and a standard deviation estimated based on the data^42,48,49^. Convergence was checked using the r-hat metric. These parameters allowed to identify the main fixed-effects as discussed in the results section.

Some of the found effects, for example the interaction between choice-history bias and participant group (*c*_1_ : *g*), were ambiguous, as they changed their direction for post-stimulation data (see Table 1). To shed light on the development of these effects over the course of a session and distinguish adaptation effects from potential tDCS stimulation effects, we further split the pre- and post-stimulation blocks in two and fitted GLMM parameters separately for each batch. Thus, each session’s data already consisting of two blocks of 98 trials each with a different tDCS stimulation between them - was split into four batches of 49 trials each, two consecutive batches in the pre-stimulation block and two in the post-stimulation block. These batches were aggregated over all sessions before fitting model parameters, resulting in four sets of parameters that coarsely capture adaptation effects over the course of a session.

Because the experiment contained different tDCS stimulation conditions, we furthermore explored model parameters fitted on the data of each individual stimulation condition to distinguish general order or adaptation effects from potential tDCS effects. This analysis confirmed previous findings that LFC-RPA stimulation leads to a change in the reliance on spatial stimulus components in SSD patients. Note that the effects of tDCS were not the focus of the present study, see Supplementary Material D for further details.

To increase reliability and trust in our findings, we fitted additional GLMMs with only a subset of the parameters reported in Eq. (3) and conducted model comparison using leave-one-out information criterion (LOOIC^50^, see also ref. ^51^) that estimates out-of-sample prediction error. Specifically, we compared models without a choice- or trial-history effect (Eq. (5)), with only a choice-history effect (Eq. (6)) and only trial-history effects (Eq. (7)) against the above reported full model (Eq. (3)). Model comparison showed that the full model provided the best overall fit. Thus, the results presented focus on the parameters from this particular model. For the full comparison see Supplementary Material A.

5 $$c\sim g\times \delta \times \alpha +(1\left|{subj}\right.)$$

6 $$c\sim g\times \delta \times \alpha \times {c}_{1}+(1\left|{subj}\right.$$

7 $$c\sim g\times \delta \times \alpha \times {\delta }_{1}\times {\alpha }_{1}+(1\left|{subj}\right.)$$

## Supplementary information

Supplementary Material

Example video

## Data Availability

The data underlying the analyses and results presented herein is available at https://osf.io/xckfb/.

## Code availability

The code created and used for statistical analyses and models is available at https://git.rwth-aachen.de/tam/choice history in causality perception and https://osf.io/xckfb/.
