# Supplementary material for: Choice- and trial-history effects on causality perception in Schizophrenia Spectrum Disorder
Source: Schizophrenia (Heidelb). 2025 Apr 17;11(1):65. doi: 10.1038/s41537-025-00614-0 (PMC12006371; doi:10.1038/s41537-025-00614-0)
Supplement: Supplementary file 1 — Supplementary Material [file 41537_2025_614_MOESM1_ESM.pdf]

Supplementary Material for Choice- and trial-history effects on causality perception in Schizophrenia

Kai Streiling[1*], Rasmus Schülke[2], Benjamin Straube[3, 4†], Loes C. J. van Dam[1, 3, 5†]

[1*]Institute for Psychology and Centre for Cognitive Science, Technical University of Darmstadt, 64283 Darmstadt, Germany .
[2]Department of Psychiatry, Social Psychiatry and Psychotherapy, Hannover Medical School, 30625 Hannover, Germany .
[3]Center for Mind, Brain and Behavior, University of Marburg and Justus Liebig University Giessen, 35032 Marburg, Germany .
[4]Department for Psychiatry and Psychotherapy, Philipps University Marburg, 35039, Marburg, Germany .
[5]Department of Psychology, University of Essex, Colchester, United Kingdom .

*Corresponding author(s). E-mail(s): kai.streiling@tu-darmstadt.de;
†These authors contributed equally to this work.

A - Model comparison

We compare models with regard to the influence of different combinations of history-effects. The following notations use the same symbols as described in the main text which are

- c - response given ("causal" or "non-causal")
- g - participant group ("HC" or "SSD")
- δ - delay of the current trial
- α - angle of the current trial
- c_1 - response given on the previous trial
- δ_1 - delay of the previous trial
- α_1 - angle of the previous trial

- $(1|subj)$ - random intercept per subject

In order to explore the relevance and interplay of choice- and trial-history effects we compare the following models:

1. No history effect

$$c \sim g \times \delta \times \alpha + (1|subj) \tag{1}$$

2. Choice-history effects with interactions (Equation 2a) or with parameters nested in the participants' group (Equation 2b)

$$c \sim g \times \delta \times \alpha \times c_1 + (1|subj) \tag{2a}$$
$$c \sim g + \delta_{HC} + +\delta_{SSD} + \alpha_{HC} + \alpha_{SSD} + c_{1,HC} + c_{1,SSD} + (1|subj) \tag{2b}$$

3. Trial-history effects with interactions (Equation 3a) or with parameters nested in the participants' group (Equation 3b)

$$c \sim g \times \delta \times \alpha \times \delta_1 \times \alpha_1 + (1|subj) \tag{3a}$$
$$c \sim g + \delta_{HC} + +\delta_{SSD} + \alpha_{HC} + \alpha_{SSD} + \delta_{1,HC} + \delta_{1,SSD} + \alpha_{1,HC} + \alpha_{1,SSD} + (1|subj) \tag{3b}$$

4. Choice- and trial-history effects with interactions (Equation 4) or with parameters nested in the participants' group (Equation 5)

$$c \sim g \times \delta \times \alpha \times c_1 \times \delta_1 \times \alpha_1 + (1|subj) \tag{4}$$
$$\begin{aligned} c \sim g + \delta_{HC} + +\delta_{SSD} + \alpha_{HC} + \alpha_{SSD} + c_{1,HC} + c_{1,SSD} \\ + \delta_{1,HC} + \delta_{1,SSD} + \alpha_{1,HC} + \alpha_{1,SSD} + (1|subj) \end{aligned} \tag{5}$$

The results of the model comparison are summed up in Table 1. The models are reported in decreasing order according to their expected log pointwise predictive density (ELPD) estimate where a higher ELPD value indicates a better out-of-sample predictive fit. We observe the strongest improvement in fit when adding the trial-history effects and their interactions to a choice-history model with interactions (2a $\rightarrow$ 4) or respectively, when adding interaction-effects to a model that already considers choice- and trial-history effects nested in the *group* variable (5 $\rightarrow$ 4). A smaller improvement can be observed when adding a choice-history effect (1 $\rightarrow$ 2a and 1 $\rightarrow$ 2b). Adding trial-history effects on their own (1 $\rightarrow$ 3a and 1 $\rightarrow$ 3b) shows no direct improvement or no relevant improvement as the estimated standard error exceeds the estimated ELPD difference (see Table 2).

We see that while there is some relevance in the choice-history and its interaction with the other parameters, the strongest improvement is gained by incorporating both, choice- and trial-history effects *and* their interaction effects. These results suggest that trial-history mainly affects the current choice by modulating other effects.

Model reference	ELPD (LOO)	SE	ELPD diff.	diff. SE	p_eff
	Pre stimulation				
4 (full model)	-10024.15	76.17	0.0	0.0	108.45
2a	-10348.22	72.95	324.06	29.76	53.55
5	-10359.10	72.77	334.95	30.45	47.48
2b	-10368.36	72.61	344.21	30.85	43.35
1	-10401.15	72.41	376.99	32.09	43.87
3b	-10401.97	72.52	377.82	32.04	48.16
3a	-10410.90	72.78	386.74	31.71	71.17

Table 1: Statistics of the model comparison for models fitted on pre-stimulation data. Models are arranged in decreasing order with regard to their expected log pointwise predictive density (ELPD) which is estimated with pareto smoothed importance sampling leave-one-out cross-validation (LOO). The ELPD estimate is reported on a logarithmic scale, which means that higher ELPD values indicate a higher out-of-sample predictive fit. Additionally, the standard error (SE) of the ELPD estimate is reported. For better comparison between models also the difference of the ELPD to the top ranked model (ELPD diff.) and the standard error of this difference (diff SE) is reported. Finally, the estimated effective number of parameters is reported (p_eff). See the arviz API documentation for more information: https://python.arviz.org/en/stable/api/generated/arviz.compare.html

Model reference	ELPD (LOO)	SE	ELPD diff.	diff. SE	p_eff
	Post stimulation				
4 (full model)	-9685.78	77.68	0.0	0.0	109.83
2a	-9894.74	75.85	208.95	24.64	53.07
5	-9911.38	75.62	225.59	25.65	47.60
2b	-9917.44	75.51	231.65	26.08	43.33
3b	-9970.60	75.37	284.82	28.06	48.21
1	-9977.38	75.27	291.59	28.42	43.86
3a	-9982.67	75.54	296.88	27.94	70.95

Table 2: Statistics of the model comparison for models fitted on post-stimulation data.

B - Pre and post stimulation model fits

Here, we provide a full list of model parameter fits on pre- and post-stimulation data from the reported models.

Model parameter fits

Table 3 shows the parameters of the model defined in Equation 4, the model mainly discussed in the main text, fitted on data from all pre-stimulation sessions (left side) and all post-stimulation sessions (right side).

	Pre stimulation				Post stimulation			
Parameter β_X	Mean	SD	CI 3%	CI 97%	Mean	SD	CI 3%	CI 97%
**0 (Intercept)**	**-0.369**	0.175	-0.679	-0.036	**-0.308**	0.170	-0.619	0.025
g **(group)**	**0.030**	0.176	-0.289	0.367	**0.119**	0.174	-0.226	0.440
α **(angle)**	**-0.580**	0.021	-0.619	-0.539	**-0.703**	0.023	-0.749	-0.661
$g : \alpha$	**-0.141**	0.022	-0.179	-0.098	**-0.226**	0.023	-0.268	-0.183
δ **(delay)**	**-0.842**	0.022	-0.887	-0.803	**-0.943**	0.024	-0.989	-0.900
$g : \delta$	**0.324**	0.023	0.280	0.365	**0.374**	0.024	0.330	0.420
$\alpha : \delta$	-0.017	0.022	-0.058	0.026	0.011	0.024	-0.034	0.054
$g : \alpha : \delta$	-0.001	0.023	-0.046	0.040	-0.043	0.025	-0.088	0.004
c_1 **(prev. choice)**	**0.194**	0.021	0.156	0.234	**0.260**	0.023	0.214	0.300
$g : c_1$	-0.062	0.022	-0.102	-0.022	0.015	0.022	-0.027	0.057
$\delta : c_1$	**0.095**	0.022	0.053	0.134	**0.072**	0.023	0.028	0.115
$g : \delta : c_1$	**-0.040**	0.022	-0.081	0.002	**-0.047**	0.023	-0.092	-0.005
$\alpha : c_1$	**0.081**	0.021	0.042	0.121	**0.037**	0.023	-0.008	0.078
$g : \alpha : c_1$	**-0.063**	0.021	-0.105	-0.024	**-0.072**	0.023	-0.112	-0.028
$\delta : \alpha : c_1$	0.064	0.023	0.019	0.104	0.125	0.024	0.081	0.170
$g : \delta : \alpha : c_1$	-0.029	0.023	-0.071	0.012	-0.037	0.025	-0.082	0.009
α_1 **(prev. angle)**	**0.010**	0.021	-0.030	0.049	**0.046**	0.023	0.004	0.087
$g : \alpha_1$	-0.049	0.021	-0.088	-0.008	-0.031	0.022	-0.071	0.010
$\delta : \alpha_1$	0.009	0.022	-0.032	0.052	-0.020	0.024	-0.063	0.028
$g : \delta : \alpha_1$	-0.036	0.022	-0.078	0.006	0.030	0.024	-0.014	0.075
$\alpha : \alpha_1$	0.066	0.022	0.024	0.107	0.036	0.023	-0.007	0.081
$g : \alpha : \alpha_1$	0.029	0.022	-0.014	0.069	0.032	0.023	-0.014	0.074
$\delta : \alpha : \alpha_1$	-0.017	0.023	-0.057	0.029	-0.029	0.025	-0.076	0.017
$g : \delta : \alpha : \alpha_1$	-0.024	0.024	-0.067	0.021	0.022	0.025	-0.022	0.071
$c_1 : \alpha_1$	0.077	0.022	0.037	0.120	0.092	0.023	0.050	0.135
$g : c_1 : \alpha_1$	0.061	0.022	0.021	0.104	0.004	0.023	-0.041	0.047
$\delta : c_1 : \alpha_1$	-0.031	0.022	-0.073	0.010	-0.070	0.024	-0.114	-0.024
$g : \delta : c_1 : \alpha_1$	0.054	0.022	0.011	0.094	0.011	0.024	-0.034	0.057
$\alpha : c_1 : \alpha_1$	**0.448**	0.023	0.402	0.489	**0.352**	0.024	0.307	0.396
$g : \alpha : c_1 : \alpha_1$	**0.219**	0.023	0.173	0.260	**0.135**	0.024	0.087	0.179
$\delta : \alpha : c_1 : \alpha_1$	0.019	0.024	-0.027	0.061	0.032	0.025	-0.014	0.080
$g : \delta : \alpha : c_1 : \alpha_1$	0.005	0.023	-0.038	0.049	-0.019	0.025	-0.067	0.027
δ_1 **(prev. delay)**	**0.028**	0.021	-0.011	0.066	**-0.000**	0.022	-0.042	0.040
$g : \delta_1$	-0.094	0.021	-0.132	-0.053	-0.061	0.022	-0.102	-0.019
$\delta : \delta_1$	0.081	0.023	0.039	0.123	0.079	0.024	0.034	0.124
$g : \delta : \delta_1$	-0.006	0.022	-0.049	0.034	-0.067	0.024	-0.111	-0.021
$\alpha : \delta_1$	0.009	0.021	-0.029	0.052	-0.020	0.024	-0.065	0.025

$g:\alpha:\delta_1$	-0.024	0.022	-0.065	0.016	-0.022	0.024	-0.065	0.023
$\delta:\alpha:\delta_1$	-0.023	0.023	-0.064	0.022	0.024	0.025	-0.024	0.072
$g:\delta:\alpha:\delta_1$	-0.024	0.023	-0.069	0.017	-0.050	0.025	-0.097	-0.002
$c_1:\delta_1$	0.056	0.021	0.018	0.097	0.063	0.023	0.020	0.106
$g:c_1:\delta_1$	-0.072	0.021	-0.114	-0.033	-0.013	0.023	-0.057	0.029
$\delta:c_1:\delta_1$	**0.266**	0.022	0.225	0.306	**0.277**	0.024	0.234	0.324
$g:\delta:c_1:\delta_1$	-0.016	0.022	-0.057	0.025	-0.033	0.024	-0.077	0.013
$\alpha:c_1:\delta_1$	-0.085	0.022	-0.126	-0.045	-0.084	0.024	-0.130	-0.041
$g:\alpha:c_1:\delta_1$	0.004	0.022	-0.036	0.046	0.004	0.023	-0.038	0.049
$\delta:\alpha:c_1:\delta_1$	-0.053	0.024	-0.096	-0.007	-0.003	0.025	-0.049	0.041
$g:\delta:\alpha:c_1:\delta_1$	0.022	0.023	-0.023	0.062	-0.010	0.025	-0.059	0.033
$\alpha_1:\delta_1$	0.037	0.022	-0.006	0.076	0.046	0.022	0.004	0.085
$g:\alpha_1:\delta_1$	-0.007	0.021	-0.045	0.035	-0.002	0.022	-0.042	0.042
$\delta:\alpha_1:\delta_1$	0.042	0.023	0.000	0.085	0.042	0.025	-0.005	0.087
$g:\delta:\alpha_1:\delta_1$	0.017	0.022	-0.024	0.058	-0.010	0.025	-0.056	0.036
$\alpha:\alpha_1:\delta_1$	0.056	0.023	0.014	0.098	0.074	0.024	0.026	0.116
$g:\alpha:\alpha_1:\delta_1$	-0.004	0.023	-0.044	0.042	0.049	0.024	0.004	0.096
$\delta:\alpha:\alpha_1:\delta_1$	-0.016	0.024	-0.057	0.033	-0.020	0.025	-0.066	0.030
$g:\delta:\alpha:\alpha_1:\delta_1$	-0.023	0.024	-0.069	0.023	0.035	0.026	-0.012	0.082
$c_1:\alpha_1:\delta_1$	0.031	0.021	-0.010	0.070	-0.004	0.022	-0.047	0.036
$g:c_1:\alpha_1:\delta_1$	0.005	0.021	-0.036	0.045	-0.018	0.022	-0.060	0.023
$\delta:c_1:\alpha_1:\delta_1$	-0.010	0.023	-0.054	0.032	-0.029	0.024	-0.075	0.017
$g:\delta:c_1:\alpha_1:\delta_1$	0.026	0.022	-0.016	0.068	-0.008	0.024	-0.057	0.034
$\alpha:c_1:\alpha_1:\delta_1$	0.025	0.023	-0.019	0.067	0.011	0.024	-0.035	0.056
$g:\alpha:c_1:\alpha_1:\delta_1$	0.012	0.023	-0.031	0.057	0.031	0.024	-0.017	0.074
$\delta:\alpha:c_1:\alpha_1:\delta_1$	0.012	0.024	-0.033	0.059	-0.004	0.025	-0.054	0.042
$g:\delta:\alpha:c_1:\alpha_1:\delta_1$	0.016	0.024	-0.030	0.061	0.006	0.025	-0.036	0.056
$\sigma(0_{subj})$	1.095	0.140	0.839	1.356	1.084	0.134	0.862	1.353
Intercepts per subject:								
$0_{subj=2,g='HC'}$	0.685	0.263	0.182	1.148	0.669	0.261	0.174	1.151
$0_{subj=5,g='HC'}$	1.623	0.265	1.132	2.117	1.352	0.265	0.853	1.860
$0_{subj=6,g='HC'}$	-0.749	0.264	-1.211	-0.236	-0.648	0.262	-1.133	-0.147
$0_{subj=9,g='HC'}$	1.063	0.262	0.590	1.559	0.836	0.267	0.348	1.367
$0_{subj=12,g='HC'}$	0.303	0.263	-0.176	0.811	-0.006	0.263	-0.504	0.477
$0_{subj=13,g='HC'}$	1.251	0.263	0.726	1.721	1.341	0.265	0.832	1.843
$0_{subj=14,g='HC'}$	-0.904	0.263	-1.385	-0.413	-0.765	0.265	-1.310	-0.314
$0_{subj=15,g='HC'}$	-0.860	0.264	-1.335	-0.361	-1.026	0.265	-1.529	-0.522
$0_{subj=17,g='HC'}$	-0.808	0.264	-1.330	-0.349	-0.935	0.267	-1.461	-0.444
$0_{subj=18,g='HC'}$	-0.243	0.260	-0.734	0.235	-0.085	0.262	-0.569	0.426
$0_{subj=21,g='HC'}$	0.765	0.261	0.290	1.249	0.454	0.261	-0.069	0.919
$0_{subj=22,g='HC'}$	0.095	0.263	-0.419	0.584	0.290	0.261	-0.199	0.790
$0_{subj=23,g='HC'}$	0.415	0.265	-0.112	0.888	0.448	0.263	-0.079	0.925
$0_{subj=24,g='HC'}$	0.603	0.260	0.116	1.078	0.434	0.263	-0.059	0.935
$0_{subj=26,g='HC'}$	-0.320	0.260	-0.804	0.173	-0.591	0.263	-1.115	-0.124
$0_{subj=27,g='HC'}$	-1.066	0.262	-1.542	-0.559	-0.936	0.266	-1.438	-0.426

$0_{subj=28,g='HC'}$	0.180	0.259	-0.304	0.657	0.163	0.262	-0.336	0.648
$0_{subj=29,g='HC'}$	-2.178	0.277	-2.679	-1.645	-2.040	0.276	-2.570	-1.544
$0_{subj=30,g='HC'}$	0.794	0.260	0.292	1.258	1.254	0.263	0.751	1.749
$0_{subj=101,g='SSD'}$	-0.358	0.274	-0.890	0.139	-0.041	0.264	-0.524	0.471
$0_{subj=102,g='SSD'}$	-0.655	0.276	-1.158	-0.130	-0.532	0.266	-1.012	-0.019
$0_{subj=103,g='SSD'}$	0.628	0.273	0.119	1.142	0.126	0.263	-0.349	0.628
$0_{subj=105,g='SSD'}$	-1.667	0.286	-2.183	-1.115	-1.929	0.282	-2.458	-1.389
$0_{subj=106,g='SSD'}$	0.925	0.284	0.415	1.468	0.797	0.267	0.290	1.287
$0_{subj=107,g='SSD'}$	-1.666	0.289	-2.207	-1.107	-1.816	0.285	-2.357	-1.286
$0_{subj=108,g='SSD'}$	-0.650	0.273	-1.170	-0.140	-0.385	0.262	-0.897	0.091
$0_{subj=109,g='SSD'}$	0.113	0.277	-0.385	0.633	-0.171	0.266	-0.668	0.329
$0_{subj=110,g='SSD'}$	0.219	0.271	-0.263	0.744	0.316	0.267	-0.189	0.835
$0_{subj=111,g='SSD'}$	0.881	0.279	0.370	1.422	0.799	0.266	0.301	1.310
$0_{subj=112,g='SSD'}$	-1.800	0.292	-2.328	-1.221	-1.404	0.273	-1.950	-0.926
$0_{subj=113,g='SSD'}$	0.794	0.268	0.269	1.288	1.157	0.271	0.640	1.669
$0_{subj=114,g='SSD'}$	0.457	0.271	-0.086	0.929	0.606	0.263	0.087	1.075
$0_{subj=115,g='SSD'}$	-0.112	0.271	-0.639	0.380	-0.168	0.264	-0.642	0.335
$0_{subj=116,g='SSD'}$	2.027	0.290	1.463	2.543	2.145	0.283	1.643	2.709
$0_{subj=117,g='SSD'}$	1.776	0.283	1.227	2.280	1.593	0.272	1.111	2.122
$0_{subj=118,g='SSD'}$	-1.797	0.292	-2.346	-1.253	-1.888	0.282	-2.439	-1.369
$0_{subj=119,g='SSD'}$	0.279	0.273	-0.261	0.761	-0.102	0.263	-0.582	0.390
$0_{subj=120,g='SSD'}$	0.643	0.277	0.132	1.149	0.834	0.267	0.324	1.325

Table 3: Full parameter list of the "full" GLMM model described in Equation 4. Reported are posterior distributions of parameter fits based on all pre-stimulation (left side) and post-stimulation (right side) data. Values of interest discussed in the main text or the Supplementary Material are marked bold.

Further interaction effects

When conducting model comparison we have seen that adding choice- and trial-history interactions leads to a significant out-of-sample predictive fit. Thus, we can expect strong interaction-effects of a higher level in the GLMM described in Equation 4, and, indeed, we find significant three-way interactions between a cue (angle or delay) in the current trial, it's trial-history (it's value in the previous trial) and the choice-history. Furthermore, we find a significant group difference of this effect for the angle (see Table 4).

	Pre stimulation				Post stimulation			
Parameter β_X	Mean	SD	CI 3%	CI 97%	Mean	SD	CI 3%	CI 97%
$\alpha : c_1 : \alpha_1$	**0.448**	0.023	0.402	0.489	**0.352**	0.024	0.307	0.396
$g : \alpha : c_1 : \alpha_1$	**0.219**	0.023	0.173	0.260	**0.135**	0.024	0.087	0.179
$\delta : c_1 : \delta_1$	**0.266**	0.022	0.225	0.306	**0.277**	0.024	0.234	0.324
$g : \delta : c_1 : \delta_1$	-0.016	0.022	-0.057	0.025	-0.033	0.024	-0.077	0.013

Table 4: Higher-level interaction-effects of interest from the pre- and post-stimulation parameter fit of the "full" interaction model (see Equation 4). These parameters are also reported in Table 3 and are reprinted here for convenience.

The data underlying these effects is shown in Fig. 4 and Fig. 5 in the main text. They show the influence of the current trial's angle (respectively delay) on the probability to respond "causal". In the figures the components of the interaction-effects summarised in Table 4 are marked in red to help localize them in the presented data.

Plots for post-stimulation fits

Effect of spatial and temporal properties

Figure 1 shows the influence of the current trial's properties angle and delay on the probability to report "causal" for the post-stimulation data. We find the effects described in the main text for the pre-stimulation data here, too.

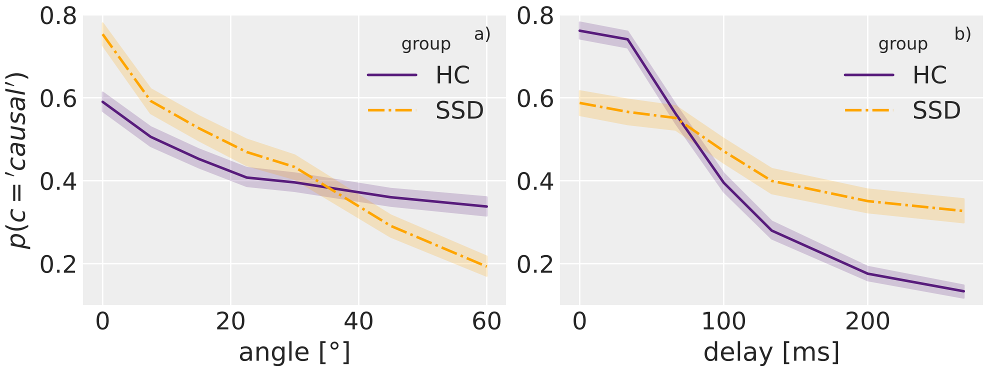

Fig. 1 The probability of reporting causal $p(c = causal)$ depending on angle (A) and delay (B), split for HCs and SSDs for the post-stimulation data. The graphs show the mean and 95 % confidence intervals calculated using a non-parametric bootstrapping approach. SSDs show a stronger dependence on spatial properties and a weaker dependence on temporal properties than HCs when reporting perceived causality.

Figure 2 shows the influence of the current trial's properties angle and delay on the probability to give the same judgement as in the previous trial ($p(c = c_1)$) for the post-stimulation data. We see that the effects described in the main text for the pre-stimulation data hold here, too.

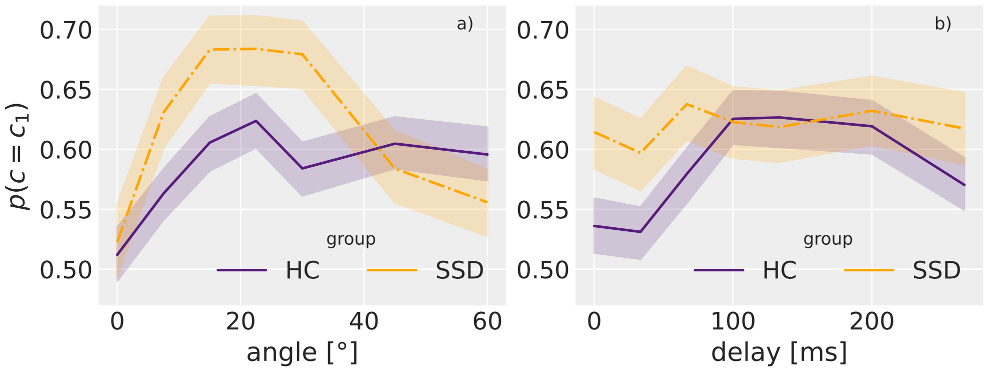

Fig. 2 The probability of reporting the same as in the previous trial $p(c = c_1)$ depending on angle (A) and delay (B), split for HCs and SSDs for post-stimulation data.

C - Parameter development

We split the data into four consecutive batches (early/ late pre-stimulation and early/ late post-stimulation) as described in the main text and fitted the "full" GLMM (see Equation 4) separately on these batches. The resulting parameter means are reported in Table 5. Figure 3 shows the development of the posterior parameter distribution for selected parameters.

	Pre-stim early		Pre-stim late		Post-stim early		Post-stim late	
	Mean	SD	Mean	SD	Mean	SD	Mean	SD
0 (intercept)	**-0.359**	0.186	**-0.344**	0.191	**-0.287**	0.179	**-0.351**	0.181
g	**0.029**	0.176	**0.004**	0.190	**0.134**	0.184	**0.055**	0.185
δ	**-0.849**	0.031	**-0.878**	0.033	**-0.888**	0.033	**-1.043**	0.036
$g:\delta$	**0.287**	0.031	**0.360**	0.032	**0.357**	0.032	**0.381**	0.036
α	**-0.570**	0.032	**-0.606**	0.032	**-0.679**	0.032	**-0.773**	0.035
$g:\alpha$	**-0.166**	0.032	**-0.114**	0.031	**-0.226**	0.032	**-0.249**	0.036
$\delta:\alpha$	**-0.016**	0.032	**-0.016**	0.033	**0.008**	0.034	**-0.019**	0.037
$g:\delta:\alpha$	**-0.012**	0.033	**0.014**	0.032	**-0.093**	0.034	**-0.015**	0.038
c_1	**0.193**	0.031	**0.187**	0.030	**0.248**	0.032	**0.299**	0.035
$g:c_1$	**-0.140**	0.031	**0.014**	0.031	**-0.012**	0.032	**0.067**	0.034
$\delta:c_1$	**0.083**	0.031	**0.097**	0.032	**0.038**	0.033	**0.120**	0.037
$g:\delta:c_1$	**-0.049**	0.032	**-0.025**	0.032	**-0.075**	0.033	**-0.004**	0.036
$\alpha:c_1$	**0.130**	0.031	**0.037**	0.031	**0.019**	0.033	**0.084**	0.035
$g:\alpha:c_1$	**-0.063**	0.031	**-0.071**	0.031	**-0.052**	0.032	**-0.077**	0.035
$\delta:\alpha:c_1$	**0.046**	0.033	**0.081**	0.033	**0.067**	0.034	**0.227**	0.038
$g:\delta:\alpha:c_1$	**-0.025**	0.032	**-0.032**	0.033	**-0.020**	0.035	**-0.046**	0.037
α_1	**0.018**	0.031	**0.020**	0.031	**0.042**	0.031	**0.067**	0.033
$g:\alpha_1$	**-0.072**	0.031	**-0.019**	0.030	**-0.052**	0.031	**-0.003**	0.033
$\delta:\alpha_1$	**-0.009**	0.031	**0.041**	0.033	**-0.046**	0.033	**0.019**	0.036
$g:\delta:\alpha_1$	**-0.032**	0.032	**-0.044**	0.033	**0.002**	0.033	**0.052**	0.035
$\alpha:\alpha_1$	**0.044**	0.033	**0.092**	0.032	**-0.004**	0.033	**0.090**	0.036
$g:\alpha:\alpha_1$	**0.076**	0.033	**-0.001**	0.032	**0.011**	0.034	**0.055**	0.036
$\delta:\alpha:\alpha_1$	**-0.062**	0.034	**0.029**	0.034	**-0.070**	0.036	**0.030**	0.038
$g:\delta:\alpha:\alpha_1$	**-0.080**	0.034	**0.014**	0.035	**-0.006**	0.036	**0.056**	0.037
$c_1:\alpha_1$	**0.096**	0.032	**0.065**	0.031	**0.147**	0.033	**0.025**	0.035
$g:c_1:\alpha_1$	**0.049**	0.032	**0.069**	0.032	**0.010**	0.032	**0.004**	0.034
$\delta:c_1:\alpha_1$	**-0.074**	0.032	**-0.002**	0.033	**-0.082**	0.033	**-0.079**	0.037
$g:\delta:c_1:\alpha_1$	**0.089**	0.033	**0.019**	0.032	**0.030**	0.034	**-0.007**	0.036
$\alpha:c_1:\alpha_1$	**0.409**	0.033	**0.495**	0.033	**0.403**	0.034	**0.287**	0.034
$g:\alpha:c_1:\alpha_1$	**0.204**	0.032	**0.239**	0.033	**0.150**	0.034	**0.111**	0.036
$\delta:\alpha:c_1:\alpha_1$	**-0.047**	0.034	**0.067**	0.035	**0.018**	0.036	**0.011**	0.038
$g:\delta:\alpha:c_1:\alpha_1$	**0.004**	0.034	**0.024**	0.034	**-0.008**	0.036	**-0.029**	0.037
δ_1	**0.045**	0.030	**0.008**	0.031	**0.029**	0.031	**-0.012**	0.034
$g:\delta_1$	**-0.117**	0.030	**-0.077**	0.030	**-0.028**	0.031	**-0.083**	0.034
$\delta:\delta_1$	**0.072**	0.034	**0.092**	0.032	**0.090**	0.033	**0.086**	0.036

$g:\delta:\delta_1$	**-0.040**	0.032	**0.024**	0.032	**-0.071**	0.034	**-0.044**	0.036
$\alpha:\delta_1$	**0.003**	0.031	**0.015**	0.032	**-0.035**	0.032	**0.007**	0.037
$g:\alpha:\delta_1$	**-0.025**	0.032	**-0.031**	0.032	**-0.022**	0.033	**-0.013**	0.036
$\delta:\alpha:\delta_1$	**-0.065**	0.033	**0.022**	0.033	**-0.025**	0.035	**0.097**	0.038
$g:\delta:\alpha:\delta_1$	**-0.040**	0.034	**-0.012**	0.032	**-0.033**	0.036	**-0.062**	0.038
$c_1:\delta_1$	**0.081**	0.031	**0.027**	0.032	**0.072**	0.032	**0.026**	0.034
$g:c_1:\delta_1$	**-0.028**	0.030	**-0.109**	0.031	**-0.003**	0.032	**-0.038**	0.034
$\delta:c_1:\delta_1$	**0.224**	0.033	**0.310**	0.033	**0.271**	0.034	**0.272**	0.036
$g:\delta:c_1:\delta_1$	**-0.027**	0.032	**0.004**	0.032	**-0.043**	0.034	**-0.026**	0.035
$\alpha:c_1:\delta_1$	**-0.090**	0.031	**-0.096**	0.033	**-0.095**	0.032	**-0.109**	0.036
$g:\alpha:c_1:\delta_1$	**-0.021**	0.031	**0.030**	0.031	**-0.010**	0.033	**0.003**	0.036
$\delta:\alpha:c_1:\delta_1$	**-0.070**	0.034	**-0.039**	0.033	**-0.017**	0.035	**-0.012**	0.038
$g:\delta:\alpha:c_1:\delta_1$	**0.024**	0.033	**0.009**	0.032	**-0.021**	0.036	**-0.021**	0.038
$\alpha_1:\delta_1$	**0.037**	0.031	**0.044**	0.031	**0.061**	0.032	**0.009**	0.034
$g:\alpha_1:\delta_1$	**0.011**	0.031	**-0.021**	0.031	**0.021**	0.031	**-0.041**	0.033
$\delta:\alpha_1:\delta_1$	**0.015**	0.032	**0.073**	0.034	**-0.020**	0.034	**0.095**	0.038
$g:\delta:\alpha_1:\delta_1$	**0.019**	0.033	**0.026**	0.033	**0.009**	0.034	**-0.044**	0.036
$\alpha:\alpha_1:\delta_1$	**0.023**	0.033	**0.089**	0.034	**0.118**	0.033	**0.022**	0.037
$g:\alpha:\alpha_1:\delta_1$	**-0.015**	0.033	**-0.002**	0.034	**0.069**	0.036	**0.009**	0.037
$\delta:\alpha:\alpha_1:\delta_1$	**0.019**	0.034	**-0.047**	0.035	**-0.042**	0.036	**-0.012**	0.039
$g:\delta:\alpha:\alpha_1:\delta_1$	**0.002**	0.034	**-0.049**	0.035	**0.039**	0.036	**0.010**	0.039
$c_1:\alpha_1:\delta_1$	**0.059**	0.030	**0.000**	0.030	**0.001**	0.031	**-0.013**	0.034
$g:c_1:\alpha_1:\delta_1$	**0.016**	0.031	**-0.003**	0.030	**-0.030**	0.032	**0.017**	0.034
$\delta:c_1:\alpha_1:\delta_1$	**-0.014**	0.032	**-0.015**	0.033	**-0.051**	0.035	**-0.008**	0.038
$g:\delta:c_1:\alpha_1:\delta_1$	**0.027**	0.032	**0.014**	0.033	**-0.030**	0.034	**0.033**	0.037
$\alpha:c_1:\alpha_1:\delta_1$	**0.009**	0.033	**0.025**	0.033	**0.012**	0.035	**0.011**	0.037
$g:\alpha:c_1:\alpha_1:\delta_1$	**0.013**	0.033	**0.024**	0.033	**0.004**	0.035	**0.078**	0.037
$\delta:\alpha:c_1:\alpha_1:\delta_1$	**0.038**	0.034	**-0.005**	0.035	**-0.021**	0.036	**0.009**	0.039
$g:\delta:\alpha:c_1:\alpha_1:\delta_1$	**0.005**	0.034	**0.054**	0.035	**-0.018**	0.036	**0.042**	0.039
$\sigma(0_{subj})$	1.111	0.145	1.128	0.146	1.087	0.144	1.120	0.140
$0_{subj=2,g='HC'}$	0.473	0.278	0.833	0.299	0.589	0.279	0.704	0.290
$0_{subj=5,g='HC'}$	1.420	0.285	1.766	0.310	1.242	0.285	1.432	0.288
$0_{subj=6,g='HC'}$	-0.791	0.286	-0.800	0.298	-0.672	0.285	-0.682	0.284
$0_{subj=9,g='HC'}$	1.186	0.285	0.830	0.294	0.836	0.277	0.784	0.287
$0_{subj=12,g='HC'}$	0.451	0.280	0.057	0.299	0.174	0.284	-0.254	0.286
$0_{subj=13,g='HC'}$	1.179	0.283	1.261	0.300	1.206	0.284	1.449	0.290
$0_{subj=14,g='HC'}$	-0.923	0.283	-0.978	0.304	-0.663	0.285	-0.937	0.282
$0_{subj=15,g='HC'}$	-0.749	0.282	-1.054	0.303	-1.128	0.289	-0.979	0.286
$0_{subj=17,g='HC'}$	-0.801	0.283	-0.896	0.299	-0.787	0.286	-1.130	0.290
$0_{subj=18,g='HC'}$	-0.394	0.279	-0.174	0.299	-0.103	0.281	-0.120	0.279
$0_{subj=21,g='HC'}$	0.757	0.276	0.687	0.303	0.423	0.282	0.438	0.283
$0_{subj=22,g='HC'}$	-0.040	0.278	0.154	0.295	0.118	0.281	0.405	0.286
$0_{subj=23,g='HC'}$	0.341	0.283	0.392	0.298	0.358	0.289	0.496	0.290
$0_{subj=24,g='HC'}$	0.614	0.283	0.506	0.294	0.447	0.283	0.375	0.282
$0_{subj=26,g='HC'}$	-0.450	0.280	-0.267	0.294	-0.629	0.282	-0.606	0.285

$0_{subj=27,g='HC'}$	-1.211	0.289	-1.014	0.300	-1.074	0.291	-0.852	0.284
$0_{subj=28,g='HC'}$	0.180	0.279	0.086	0.295	0.229	0.280	0.050	0.286
$0_{subj=29,g='HC'}$	-2.004	0.307	-2.421	0.321	-1.847	0.300	-2.283	0.315
$0_{subj=30,g='HC'}$	0.584	0.279	0.954	0.299	1.112	0.286	1.373	0.290
$0_{subj=101,g='SSD'}$	-0.535	0.308	-0.248	0.309	-0.248	0.300	0.187	0.301
$0_{subj=102,g='SSD'}$	-0.588	0.310	-0.736	0.318	-0.584	0.308	-0.471	0.301
$0_{subj=103,g='SSD'}$	0.954	0.308	0.364	0.310	0.313	0.308	-0.050	0.301
$0_{subj=105,g='SSD'}$	-1.895	0.329	-1.473	0.329	-1.862	0.334	-1.941	0.341
$0_{subj=106,g='SSD'}$	0.939	0.315	0.913	0.317	0.706	0.310	0.926	0.308
$0_{subj=107,g='SSD'}$	-1.556	0.325	-1.791	0.347	-1.978	0.346	-1.638	0.320
$0_{subj=108,g='SSD'}$	-0.915	0.313	-0.456	0.309	-0.225	0.299	-0.493	0.307
$0_{subj=109,g='SSD'}$	0.049	0.301	0.130	0.309	-0.125	0.302	-0.202	0.309
$0_{subj=110,g='SSD'}$	0.359	0.304	0.080	0.308	0.177	0.301	0.475	0.305
$0_{subj=111,g='SSD'}$	0.834	0.308	0.908	0.318	0.856	0.313	0.755	0.306
$0_{subj=112,g='SSD'}$	-2.029	0.343	-1.603	0.333	-1.212	0.310	-1.582	0.326
$0_{subj=113,g='SSD'}$	0.684	0.304	0.857	0.308	1.223	0.306	1.115	0.315
$0_{subj=114,g='SSD'}$	0.617	0.302	0.305	0.308	0.626	0.303	0.586	0.306
$0_{subj=115,g='SSD'}$	-0.217	0.304	-0.034	0.308	-0.085	0.301	-0.226	0.306
$0_{subj=116,g='SSD'}$	1.945	0.322	2.131	0.338	2.167	0.341	2.086	0.332
$0_{subj=117,g='SSD'}$	1.858	0.325	1.695	0.325	1.536	0.317	1.657	0.323
$0_{subj=118,g='SSD'}$	-1.737	0.331	-1.853	0.342	-2.050	0.346	-1.705	0.325
$0_{subj=119,g='SSD'}$	0.483	0.299	0.076	0.310	-0.167	0.299	-0.018	0.307
$0_{subj=120,g='SSD'}$	0.323	0.310	0.931	0.309	0.950	0.307	0.755	0.307

Table 5: Parameter development of the GLMM described in Equation 4. Reported are posterior mean (marked in bold) and standard deviation SD for the model fits on early and late pre-stimulation and early and late post-stimulation data.

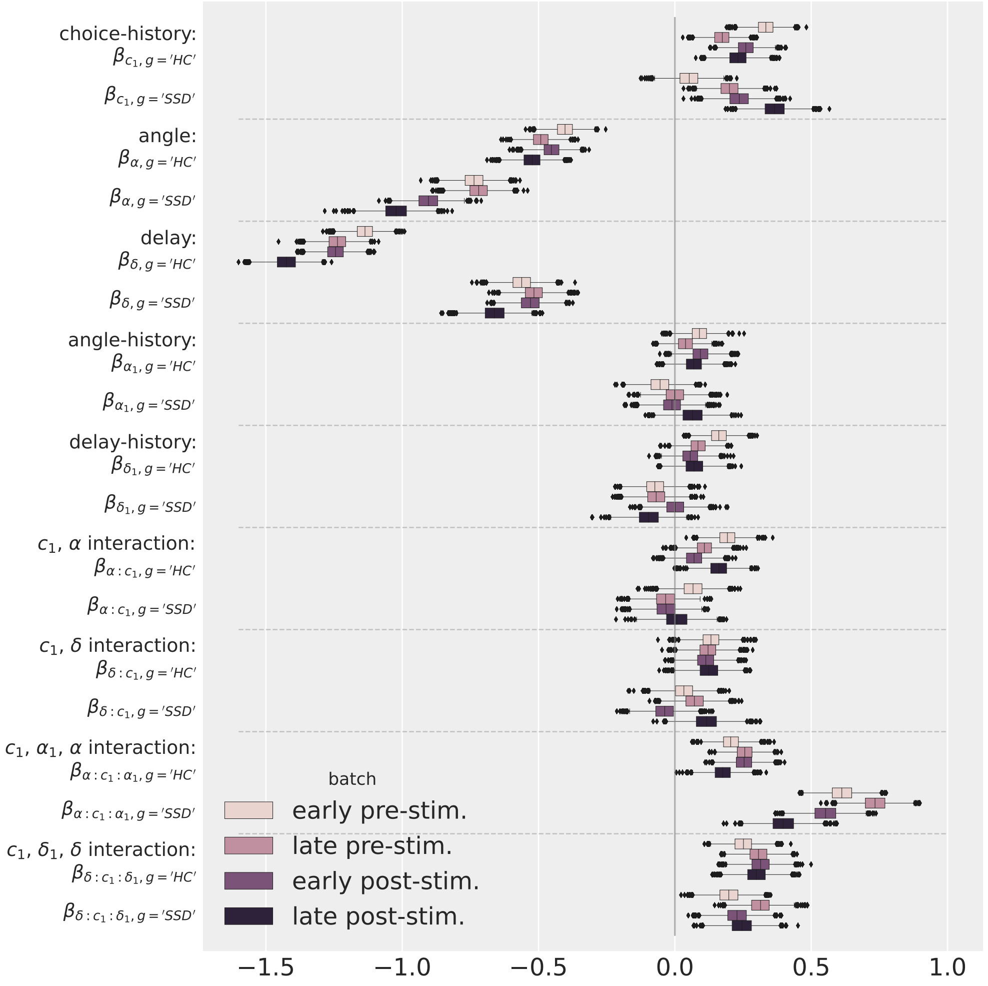

Fig. 3 Posterior distribution of parameters split by participant group (HC and SSD). The reported parameters are the sum of the base parameters and the respective interaction. For example, $\beta_{c_1,g} = \beta_{c_1} + g \cdot \beta_{g:c_1}$, $g = 1$ if SSD, $g = -1$, if HC. Each box shows the mean and the 25 % and 75 % quartile. The whiskers include datapoints within 1.5 times IQR (inter-quartile range). Any points outside are drawn as dots. See also the description in the main text for more information.

D - tDCS analysis

The original experiment investigated the effect of transcranial direct current stimulation (tDCS) at different locations on perceptual causality judgements of launching events. The initial analysis considered only data for SSDs and reported a tDCS effect of fronto-parietal stimulation (left-frontal cathodal to right-parietal anodal) on the angle-effect [1]. Here, we also examined tDCS modulations of angle and delay effects for HCs and of choice- and trial-history effects for both groups.

Following the analysis in [1], we do pairwise comparison between pre- and post-stimulation data for each stimulation condition for each value of *angle*, *delay* and respectively *choice-history*.

As can be seen in Figures 4, 5 and 6, the only condition leading to consistent significant differences across multiple values of the applied variable range was the previously reported LFC-RPA stimulation on the *angle* effect for SSDs. We found no consistent effects for HCs and none on the influence of choice-history.

We further checked for effects of tDCS on the parameter development reported in the main text and Supplementary Material Section C. We excluded the LFC-RPA stimulation session from our data and reran the analysis described above (see Section C). All effects described in the main text still held. The most distinct difference could be observed in angle-related effects between the pre- and post-stimulation fits (e.g. the jump from pre- to post-stimulation fits in $\beta_{\alpha,g='SSD'}$ in Figure 3 was less pronounced). As already described in the main text, this further indicates that these parameter shifts captured the effect of the tDCS stimulation.

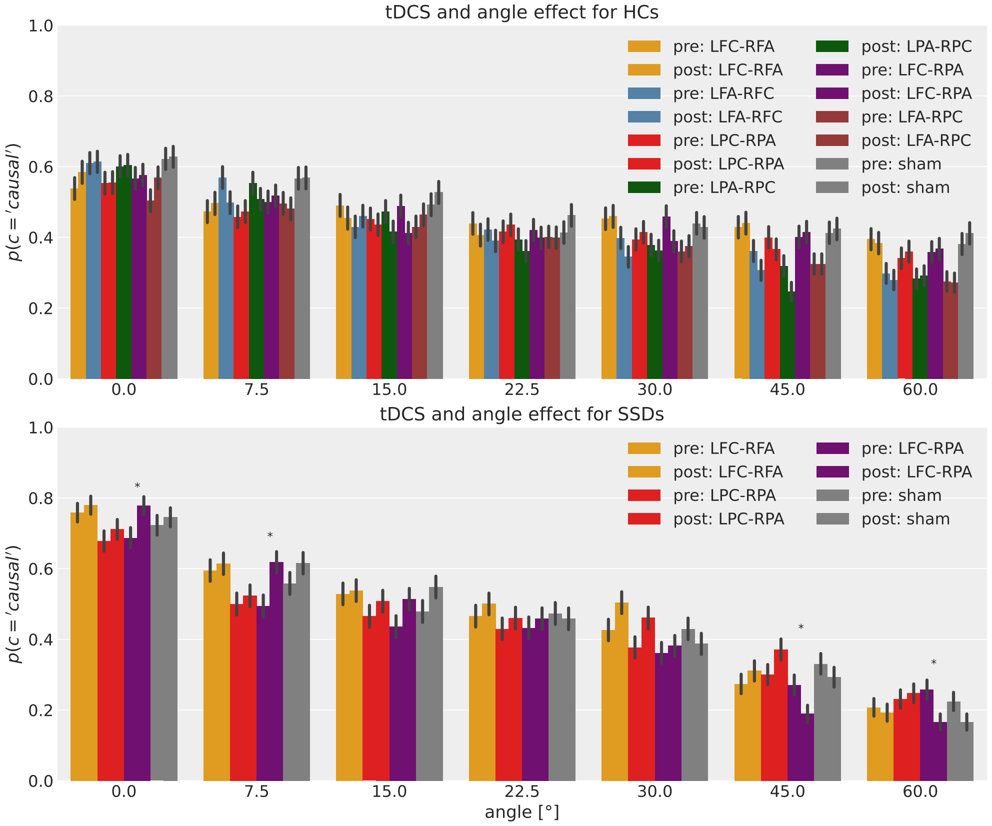

Fig. 4 Data comparison between pre- and post-tDCS sessions for different angle values. The y-axis displays the probability to report "causal". On the x-axis, for each value of angle, for each applied tDCS condition, the pre- and post-stimulation data is shown. Data for HCs is presented in the upper panel, data for SSDs in the lower. Significant differences - according to a p-value of less than 5 % - between pre- and post-stimulation data are marked with *.

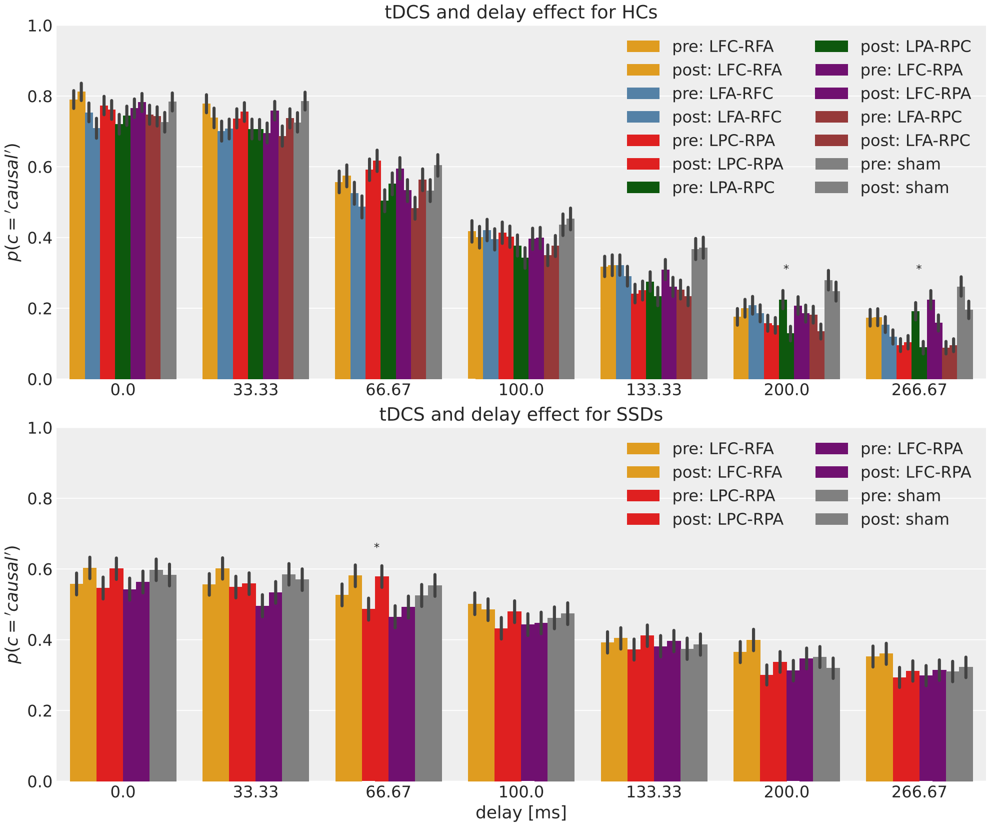

Fig. 5 Data comparison between pre- and post-tDCS sessions for different delay values. The y-axis displays the probability to report "causal". On the x-axis, for each value of delay, for each applied tDCS condition, the pre- and post-stimulation data is shown. Data for HCs is presented in the upper panel, data for SSDs in the lower. Significant differences - according to a p-value of less than 5 % - between pre- and post-stimulation data are marked with *.

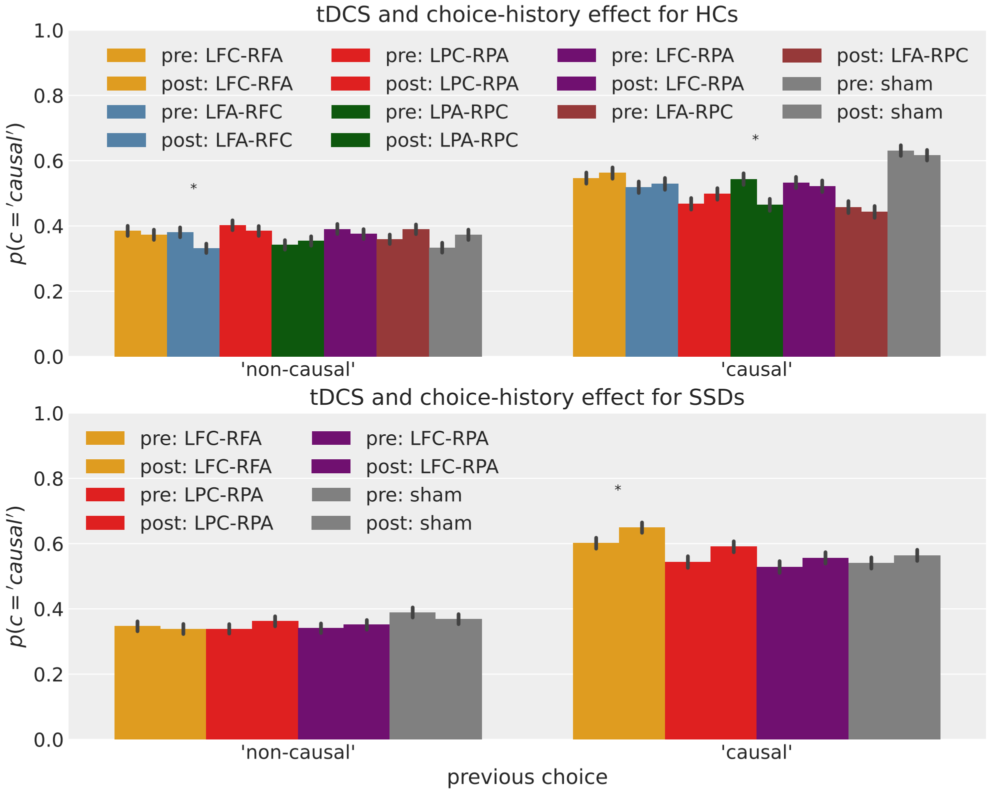

Fig. 6 Data comparison between pre- and post-tDCS sessions for different choice-history values. The y-axis displays the probability to report "causal". On the x-axis, for each possible previous judgement, for each applied tDCS condition, the pre- and post-stimulation data is shown. Data for HCs is presented in the upper panel, data for SSDs in the lower. Significant differences - according to a p-value of less than 5 % - between pre- and post-stimulation data are marked with *.

E - Participant group comparison

The original experiment collected data from 20 participants with diagnosed SSD and further measured 20 neurotypical participants matched according to basic demographic criteria as a control group. For the analysis one participant from each group was dropped, leaving 19 SSDs and 19 HCs. Table 6 summarizes their basic demographic data, positive and negative SSD symptoms (SAPS/SANS), performance on cognitive tests performed prior to the first experiment session, and custom questions asked after the first session.

The comparison shows that both groups are well comparable. They did not differ significantly on any of the demographic measures. Further, they showed non-different performance on the Digit Span test (DS) which indicates working memory capacity, the vocabulary test (MWT-B) and the the trail making test B (TMT-B).

	SSD				HC				Comparison		
	n	Mean ± SD	Med	Min/Max	n	Mean ± SD	Med	Min/Max	T	p	d
Femal/Male	2/17				2/17						
Age	19	39.6 ± 11.2	39	20/61	19	40.1 ± 13.3	36	22/60	0.1	0.9	0.04
Education level	19	5.6 ± 2	6	2/9	19	5.6 ± 2.2	5	3/9	-0.1	0.9	-0.03
lower secondary	3				4						
upper secondary	14				10						
tertiary	2				5						
years of edu.	18	14 ± 4.6	14.5	0.5/22	19	16.5 ± 4	15	11/25	1.8	0.1	0.6
SAPS	17	10.5 ± 12.6	7	0/50							
SANS	17	17.6 ± 17.7	13	0/57							
MWT-B	19	30.6 ± 3.7	32	21/36	19	31.5 ± 3.7	31	21/37	0.7	0.5	0.2
TMT-A	19	32.3 ± 9.7	31	19/57	19	24.3 ± 4.9	25	15/32	-3.2	0.003	-1.1
TMT-B	18	62.4 ± 19.7	56	38/115	19	55.5 ± 12.2	53	36/77	-1.3	0.2	-0.4
DSST	19	43.6 ± 8.5	45	22/55	19	56.2 ± 9.1	55	40/72	4.4	0.0001	1.5
DS	19	12.6 ± 2.3	13	8/16	19	14 ± 2.7	14	9/19	1.7	0.1	0.6
CPZ					18	583 ± 412.4	517.4	0/1390			

Table 6 Demographic data and cognitive tests results compared between participants with and without SSD. Comparison was done using independent-samples t-tests of which the t-statistic, the p-value and Cohen's d are reported. The education level was measured according to the CASMIN system, additionally years of education are reported. The reported tests are the following: SAPS - Scale for the Assessment of Positive Symptoms, SANS - Scale for the Assessment of Negative Symptoms, MWT-B - Multiple Choice Vocabulary Test B (German: *Mehrfachwahl-Wortschatz-Intelligenztest*, TMT-A/B - Trail-Making-Test A/B, DSST - Digit Symbol Substitution Test, DS - Digit Span test, CPZ - medication in Chlorpromazine equivalents.

	SSD		HC		Comparison		
	Mean ± SD	Median	Mean ± SD	Median	T	p	d
I understood the task	4.6 ± 0.6	5	4.6 ± 0.6	5	-0.3	0.8	-0.1
Sufficient time	4.2 ± 1	4.5	4.5 ± 0.5	5	1.4	0.2	0.5
Mistakes	3.4 ± 1.1	4	2.5 ± 1	2	-2.5	0.02	-0.8
Answer certainty	3.4 ± 1.1	4	3.7 ± 0.9	4	0.8	0.4	0.3
Concentration	3.5 ± 1	4	3.8 ± 0.9	4	0.9	0.4	0.3

Table 7 Answers collected after the first session compared between participants with and without SSD. Comparison was done using independent-samples t-tests of which the t-statistic, the p-value and Cohen's d are reported. The full-length questions in the same order as shown in the table were: *I understood the ball-video task.*, *After the ball videos I had enough time for my decision and button press.*, *In the ball videos, I often pushed the wrong button accidentally.*, *I could still concentrate sufficiently by the end of the experiment (ball videos).*, *With most ball videos I was certain about my answer. I could still concentrate sufficiently by the end of the experiment (button press).*

After the first session participants were asked multiple questions to assess their involvement in the study. A selection of their answers is displayed in Table 7 again split and compared between HCs and SSDs. We see that both groups reported that they understood the task, had sufficient time to give their response, were certain about their answer and could still concentrate by the end of the session. The groups differed in their retrospective self-assessment of errors they made though. SSDs reported that they thought they had pressed the wrong button more often.

F - Frequentist analysis of main findings

The main text presents a Generalised-Linear-Mixed-Effects Model (GLMM) fitted using Bayesian statistics. This approach allowed for a comprehensive analysis and helped identifying higher-level interaction effects between parameters. However, to support comparability and further underline the statistical credibility of our findings also to a Frequentist audience, we here additionally provide Frequentist based approaches confirming our main findings. The corresponding code is available at https://osf.io/xckfb/.

As participants' answers were binary ("non causal" $\equiv 0$, "causal" $\equiv 1$) and aggregated results per participant represent proportions, we used non-parametric tests for within (Wilcoxon signed-rank test and Friedman test) and between group (Mann-Whitney-U) comparisons. One reason why Frequentist statistics are not a good match for the current work is that these tests do not allow comparisons across more than one factor at a time (there are no 2-way or n-way variants), meaning that interaction terms are hard to identify. The reported p-values were not corrected for multiple comparisons (e.g. using Bonferroni correction) to account for the difference in sensitivity with Bayesian statistics. Statements about significance are made in relation to a significance level of $\alpha = 0.05$. The following section lists our main findings from Section 2 of the main text and explains one approach towards replicating them using Frequentist based tests for each.

Section 2.1 and 2.2 - Angle, delay and the previous choice had an influence on reporting perceiving causality.

As reported in Table 1 in the main text, our GLMM reports that the 94% credibility interval of the posterior distributions of the effects for angle, delay and previous choice do not contain 0, which marks these effects as *considerable*. These effects are further reported to be stronger for the post-stimulation fit compared to the pre-stimulation fit.

To test these effects using a Frequentist approach, for angle and delay separately, we performed a Friedman test on the per-participant aggregated proportions of causal answers per angle and delay value. For both variables, the test reported a significant influence (angle: $\chi^2 = 51.5883$, $p < 0.0001$; delay: $\chi^2 = 73.9273$, $p < 0.0001$) for the whole population. Separating HCs and SSDs and testing separately, resulted for HCs in significant effects of angle ($\chi^2 = 15.0412$, $p = 0.0199$) and delay ($\chi^2 = 79.9431$, $p < 0.0001$) and for SSDs in a significant effect of angle ($\chi^2 = 43.6507$, $p < 0.0001$) but no significant effect of delay ($\chi^2 = 11.1973$, $p = 0.0825$). As a post-hoc test a pairwise comparison of the proportions of causal answers between the different angle (delay) values using a Wilcoxon signed-rank test was performed. For the whole population as well as for both groups, especially the difference between the most extreme value pairs (angle 0 to 60 °, delay 0 to 267 ms) was significant (whole population: between 60°and 0° angle: $W = 85$, $p < 0.0001$, between 267 ms and 0 ms delay: $W = 38$, $p < 0.0001$, HCs: between 60°and 0° angle: $W = 39$, $p = 0.023$, between 267 ms and 0 ms delay: $W = 1$, $p < 0.0001$, SSDs: between 60°and 0° angle: $W = 13$, $p < 0.0003$, between 267 ms and 0 ms delay: $W = 28$, $p = 0.0123$). For the effect of the previous choice a within subject comparison using the Wilcoxon signed-rank test was performed. For the whole population the test reported a significant difference between the previous choice being "casual" vs being "non-causal" ($W = 168$, $p = 0.0027$). For HCs only, the difference was non-significant ($W = 47$, $p = 0.0546$), for SSDs only, it was significant ($W = 42$, $p = 0.0323$). Above reported results were based on pre-stimulation data and remain consistent when tested on post-stimulation data.

Section 2.1 - The influence of angle and delay differed between HCs and SSDs.

The GLMM reported a considerable group-angle and a group-delay interaction-effect, meaning the effects of angle and delay on reporting perceiving causality differed between HCs and SSDs. We thus needed to test for a difference between HCs and SSDs in the trend of the proportion of causal answers across angle or delay values. Assuming a linear relation, we could compare the difference in the proportion of causal responses between the extreme values (e.g. 60 ° and 0 ° for angle, 0 ms and 267 ms for delay) between the two groups (HCs and SSDs). A Mann-Whitney U test between the groups reported a significant difference for the effect of angle ($U = 108$, $p = 0.0356$) and delay ($U = 89$, $p = 0.0079$). These results were based on pre-stimulation data and remain significant when tested on post-stimulation data.

Section 2.3 - The current trial's angle modulated the effect of the previous choice for SSDs.

Section 2.3 describes an interaction effect between the current angle and the previous choice in their influence on reporting "causal". This effect furthermore differed between SSDs and HCs. To test for a choice-history effect in the data directly, the proportion of repeated answers between two consecutive trials was evaluated. Thus, the following tests do not test for differences in the proportion of "causal" answers but in the proportion of repeated answers. When testing for these two effects (interaction effect and a group difference), we are answering the following two questions: First, is there a significant difference in the proportion of repeated answers between different values of angle for HCs and SSDs separately? Second, is this difference significantly different for HCs and SSDs?

The first question could be answered by again performing a Friedman test and pairwise comparison of the portions of repeated answers across the angle values. The Friedmann test reported a significant influence for SSDs ($\chi^2 = 15.0538$, $p = 0.0198$), but not for HCs ($\chi^2 = 9.2573$, $p = 0.1596$). Additionally, pairwise comparison suggested a non-linear relation for SSDs as differences between angle values at the end of the applied spectrum (0° or 7.5° and 45° or 60°) and the middle values (15°, 22.5° and 30°) were significant (Wilcoxon signed-rank test, e.g. 0° to 15°: $W = 39$, $p = 0.023$; 0° to 22°: $W = 24$, $p = 0.0028$; 0° to 30°: $W = 41.5$, $p = 0.0289$) while differences between the two ends of the spectrum were not significant (e.g. 0° to 60°: $W = 70$, $p = 0.4997$). To answer the second question, we could again compare the differences in the proportions of repeated answers between different angle values across groups using a Mann-Whitney U test as we have done before. Doing so results in a significant group difference with regard to the differences between the largest angle values and the middle angle values (15° to 45°: $U = 109$, $p = 0.0382$; 15° to 60°: $U = 90$, $p = 0.0086$; 30° to 45°: $U = 111$, $p = 0.0439$; 30° to 60°: $U = 96$, $p = 0.0142$).

Section 2.5 - The choice-history effect developed differently for HCs and SSDs.

To test for a development of the choice-history effect over the course of an experiment session in our data, we could look at the change in the proportion of repeated answers. First, the data was separated into four batches as described in the main text, then, per participant and batch, the proportion of repeated answers was calculated. These proportions can be compared across batches within each group (HCs and SSDs) to test for the presence of a development in the choice-history effect separately. Furthermore, we can again take differences between the batches per participant and compare these differences between the two groups to test whether a potential development of the choice-history effect differs between HCs and SSDs.

A Friedman test on the proportion of repeated answers across batches revealed no significant development for both groups (HCs: $\chi^2 = 5.0842$, $p = 0.1657$, SSDs: $\chi^2 = 3.3790$, $p = 0.3368$). Pairwise comparison using a Wilcoxon signed-rank test between each batch reported also no significant differences for SSDs. For HCs, the test reported a significant difference between the post-stimulation blocks (post 1 - post 2: $W = 41$, $p = 0.0289$) and a trend across the whole session (pre 1 - post 2: $W = 46$,

$p = 0.0493$).
Comparing the differences between batches across groups with a Mann-Whitney U test, we found significant differences in the overall development (pre 1 - post 2: $U = 105.0$, $p = 0.0286$) and in the development during the pre-stimulation block (pre 1 - pre 2: $U = 96.0$, $p = 0.0142$).
Taken together, these results suggest that while viewed separately, neither group showed a significant development of the choice-history effect, yet when taken together the choice-history effect developed differently for both groups.

References

[1] Schülke, R., Schmitter, C.V., Straube, B.: Improving causality perception judgments in schizophrenia spectrum disorder via transcranial direct current stimulation. Journal of Psychiatry & Neuroscience : JPN **48**(4), 245–254 (2023) https://doi.org/10.1503/jpn.220184 . Accessed 2023-09-13
